# Cathelicidin LL37-loaded extracellular vesicles from *Edwardsiella piscicida* promote antibacterial and wound-healing activity

**DOI:** 10.1038/s41598-025-28377-9

**Published:** 2025-12-02

**Authors:** Mawalle Kankanamge Hasitha Madhawa Dias, E. H. T. Thulshan Jayathilaka, Mahanama De Zoysa

**Affiliations:** College of Veterinary Medicine and Research Institute of Veterinary Medicine, Chungnam National University, Yuseong-gu, Daejeon, 34134 Republic of Korea

**Keywords:** Antimicrobial peptide, Bacterial extracellular vesicles, Cathelicidin (LL37), *Edwardsiella piscicida*, *Ep*EVs-LL37, Wound-healing, Antimicrobials, Bacteria, Pathogens, Drug delivery

## Abstract

Bacterial extracellular vesicles (BEVs) have emerged as a promising therapeutic platform, offering the potential to be engineered for targeted molecular delivery. In this study, the human cathelicidin antimicrobial peptide (LL37) was encapsulated into extracellular vesicles derived from the fish pathogen *Edwardsiella piscicida* (*Ep*EVs) through co-incubation. LL37-encapsulated *Ep*EVs (*Ep*EVs-LL37) were isolated by ultracentrifugation and characterized with a mean particle size of 73.6 ± 1.4 nm and a zeta potential of -11.27 ± 0.49 mV. Morphology of *Ep*EVs-LL37 was confirmed as spherical-shaped particles. Enzymatic stability evaluation results revealed a proportionate increase in mean size and zeta potential with the pepsin concentration (0.4-2 mg/mL) at pH 2.0. *Ep*EVs-LL37 exhibited lower toxicity than free LL37 in both Raw 264.7 cells and zebrafish larvae, while maintaining a similar level of cellular internalization as naïve *Ep*EVs. Notably, *Ep*EVs-LL37 demonstrated enhanced antibacterial activity against *E. piscicida* compared to free LL37, as evidenced by time-kill kinetics and bacterial viability assays. Mechanistically, *Ep*EVs-LL37 damaged the bacterial cell membrane and induced higher levels of reactive oxygen species (ROS), along with bacterial membrane permeability, as observed through confocal microscopy. Additionally, *Ep*EVs-LL37 upregulated the expression of immunomodulatory genes (*Tlr2*,* Il1β*,* Il10*) in both Raw 264.7 cells and zebrafish larvae. Furthermore, treatment with *Ep*EVs-LL37 (10 µg/mL) significantly enhanced wound-healing in vitro and in vivo, reducing the cellular wound area to 11.01 ± 0.81% and increasing larval fin regeneration to 1.64 ± 0.07 mm², both outperforming free LL37. Collectively, these findings highlight *Ep*EVs-LL37 as a promising therapeutic and drug-delivery platform for the treatment of *E. piscicida*-associated infections.

**Supplementary Information:**

The online version contains supplementary material available at 10.1038/s41598-025-28377-9.

## Introduction

Prokaryotic and eukaryotic organisms release small membrane-bound nanostructures known as extracellular vesicles (EVs), which play multifunctional roles in maintaining cellular integrity and communication^1^. Bacterial extracellular vesicles (BEVs) are a progressing field of study due to their vast potential applications in cancer immune therapy and vaccines^2^. Both Gram-negative and Gram-positive bacteria release BEVs, which facilitate cell-cell communication, quorum sensing, biofilm formation, horizontal gene transfer, and cargo delivery functions, which are essential for bacterial infection and survival^3^. Structural components of Gram-negative BEVs comprise lipid membrane, membrane-bound or plasma proteins, nucleic acids, and lipopolysaccharide (LPS)-like toxins^3^. Due to being small nanoscale vesicles surrounded by a lipid membrane, BEVs are prime candidates for delivering virulence factors and toxins into host cells that can accelerate the infection process, aiding bacterial survival and multiplication. However, recent efforts have focused on enhancing their capacity to deliver specific molecules to target cells, positioning them as promising candidates for advanced drug delivery systems^4^. Cargo inside BEVs can be utilized as immune stimulants and disease markers to identify and strengthen host defenses^5^. Furthermore, due to their physicochemical properties, BEVs can easily reach inaccessible targets like the blood-brain barrier and lung tissues, making them ideal for being utilized as modified drug delivery agents.

Bioengineering BEVs have become one of the key research interests over the past few decades due to their biocompatibility, cargo stability, native immune-stimulating ability, cellular absorption, prolonged circulation, ex vivo upscaling, and targeted delivery^6^. Manipulations could be done to the external structure (endotoxin removal by detergent-extraction) or internal cargo, as well as to the releasing bacteria (to increase vesiculation), depending on the intended use and application^6^. Drug loading into EVs not only protects the cargo from degradation but also facilitates targeted delivery, enhanced cellular uptake, and controlled release^7^. At present, different active and passive methods are followed to load drugs into BEVs, including electroporation, extrusion, sonication, freeze-thaw cycles, chemical conjugation, co-incubation, cloaking, and genetic manipulation of releasing bacteria^6^. Utilizing these methods, therapeutic agents such as antimicrobial peptides (AMPs), small molecules, nucleic acids, or proteins can be successfully loaded. Moreover, to combat rising antimicrobial resistance, antibiotics (levofloxacin and gentamycin) have been loaded into *Acinetobacter baumannii* and *Pseudomonas aeruginosa*-derived BEVs, increasing their antibacterial capability^8,9^. Therefore, disguising antibacterial agents in BEVs can be a holistic approach to minimize antimicrobial resistance, as it provides efficient uptake and release of BEVs loaded with antimicrobial therapeutics.

*Edwardsiella piscicida* is a well-known fish pathogen that primarily enters its host through the gastrointestinal tract, causing edwardsiellosis and leading to significant economic losses in the global aquaculture industry. To manage this disease, the U.S. Food and Drug Administration recommends administering florfenicol at a dosage of 10–15 mg per kg of fish body weight for a maximum duration of 10 consecutive days^10^. However, recent studies have raised concerns that exposure to commercial antibiotics in aquaculture leads to alterations in gut microbiota and the accumulation of antibiotic-resistant genes^11,12^. AMPs are short-chain cationic, amphipathic peptides that typically consist of < 50 amino acids and exhibit broad-spectrum antimicrobial activity^13,14^. LL37 is one such AMP derived from human cathelicidin that has multiple antimicrobial activities against different Gram-negative as well as Gram-positive pathogenic bacteria^15^. However, its activity against the genus *Edwardsiella* has not yet been reported.

In our prior publication, we successfully isolated and characterized *Edwardsiella piscicida*-derived EVs (*Ep*EVs), analyzing their proteomic composition as well as in vitro and in vivo immunomodulatory effects^16^. In this study, we aim to elucidate and compare the potential antibacterial effects of LL37 encapsulated in *Ep*EVs (*Ep*EVs-LL37) against *E. piscicida*. Furthermore, we evaluate *Ep*EVs-LL37’s immunomodulatory and wound-healing activity to determine *Ep*EVs’ suitability as a drug delivery candidate.

## Results

### Physicochemical and morphological characteristics and enzymatic stability of *Ep*EVs-LL37

**Fig. 1:**
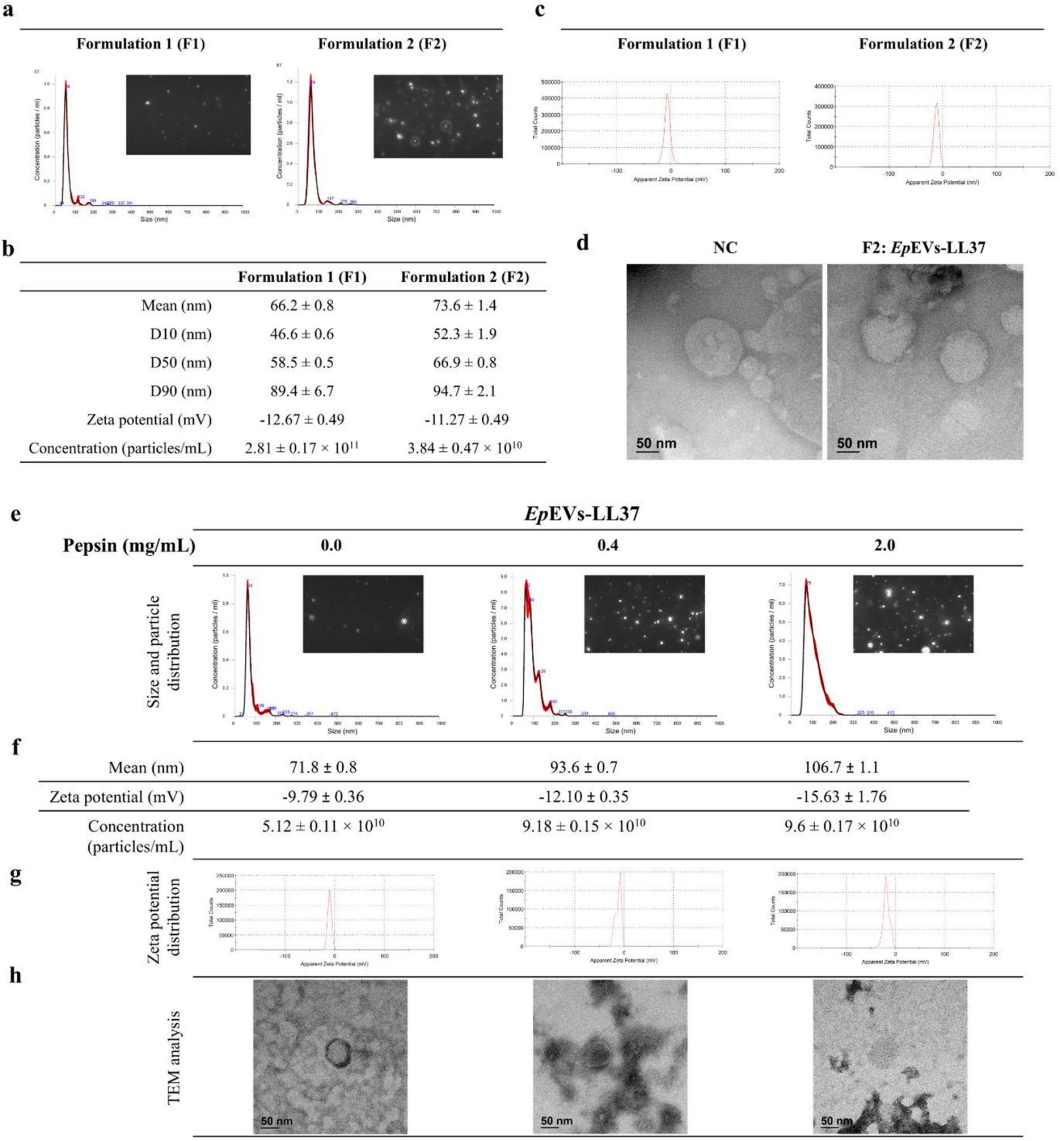
Characterization and stability of *Ep*EVs-LL37. (**a**) Particle size distribution of *Ep*EVs-LL37 was determined by nanoparticle tracking analysis (NTA), with a representative single-frame capture for both formulation 1 (F1) and formulation 2 (F2). (**b**) Quantitative comparative analysis of mean particle size and distribution, zeta potential, and particle concentration of *Ep*EVs-LL37 (F1 and F2). (**c**) Surface charge (zeta potential) distribution of *Ep*EVs-LL37. (**d**) Field emission transmission electron microscopy (FE-TEM) images of *Ep*EVs-LL37 (F2), confirming vesicle morphology. (**e**) Particle size distribution of *Ep*EVs-LL37 treated with varying concentrations of pepsin with representative NTA frame captures. (**f**) Quantitative analysis of mean particle size, zeta potential, and particle concentration of pepsin-treated *Ep*EVs-LL37. (**g**) Surface charge distribution of pepsin-treated *Ep*EVs-LL37. (**h**) FE-TEM image of the pepsin-treated *Ep*EVs-LL37, showing morphological integrity post-treatment. Data are represented as mean ± SEM. (NC - Negative control).

Table 1 shows the encapsulation efficiency (EE%) of the F1 and F2 formulations of *Ep*EVs-LL37. The F2 formulation resulted in higher EE (61.67%) compared to the F1 formulation, which was nearly half (32.67%) the EE of F2. Figure 1a shows the particle size distribution of *Ep*EVs-LL37 obtained from F1 and F2 formulations. According to our analysis, no notable difference was observed in the distribution pattern for either formulation. Furthermore, the single frame captured during the nanoparticle tracking analysis (NTA) revealed evenly distributed circular-shaped particles for both F1 and F2 formulations. The mean particle size of F1 (66.2 ± 0.8 nm) was marginally smaller compared to F2 (73.6 ± 1.4 nm) (Fig. 1b). Zeta potential of F2 (-11.27 ± 0.49 mV) was less negative compared to F1 (-12.67 ± 0.49 mV) formulation which could be due to the presence of positively charge LL37 (Fig. 1b). However, the membrane charge distribution curve showed similar pattern for both F1 and F2 formulations (Fig. 1c).

**Table 1:** Encapsulation efficiency (EE%) of *Ep*EVs-LL37.

Description	Formulation 1 (F1)	Formulation 2 (F2)
Ratio BEVs: AMPs	1:1	1:2
EE (%)	32.67	61.67

*AMP*: antimicrobial peptide, *BEVs*: bacterial extracellular vesicles.

Similar spherical shape morphology of *Ep*EVs-LL37 was observed from the field emission transmission electron microscopy (FE-TEM) images, suggesting that the encapsulation process does not affect the morphology of naïve *Ep*EVs used in this study (Fig. 1d). Based on the results, *Ep*EVs-LL37 produced using the F2 formulation was selected for further studies, as it had higher EE% with minimal changes to their morphological and physicochemical properties.

The stability of *Ep*EVs-LL37 was observed with the degradation due to stomach enzyme pepsin. The particle distribution curve displayed a slight rightward shift suggesting a mean particle size proportionately increasing with the pepsin concentration (Fig. 1e). Mean particle size was highest (106.7 ± 1.1 nm) for 2 mg/mL pepsin treated samples compared to the size of the non-treated samples (71.8 ± 0.8 nm) (Fig. 1f). Zeta potential also increased with pepsin treatment showing the highest value for 2 mg/mL pepsin treatment (-15.63 ± 1.76 mV) although membrane charge distribution for all treatments appeared similar (Fig. 1g). However, clear membrane margins with spherical shape was observed for all treatments suggesting the basic structure and integrity of *Ep*EVs-LL37 was not compromised (Fig. 1h).

### Toxicity and cellular internalization of *Ep*EVs-LL37

**Fig. 2:**
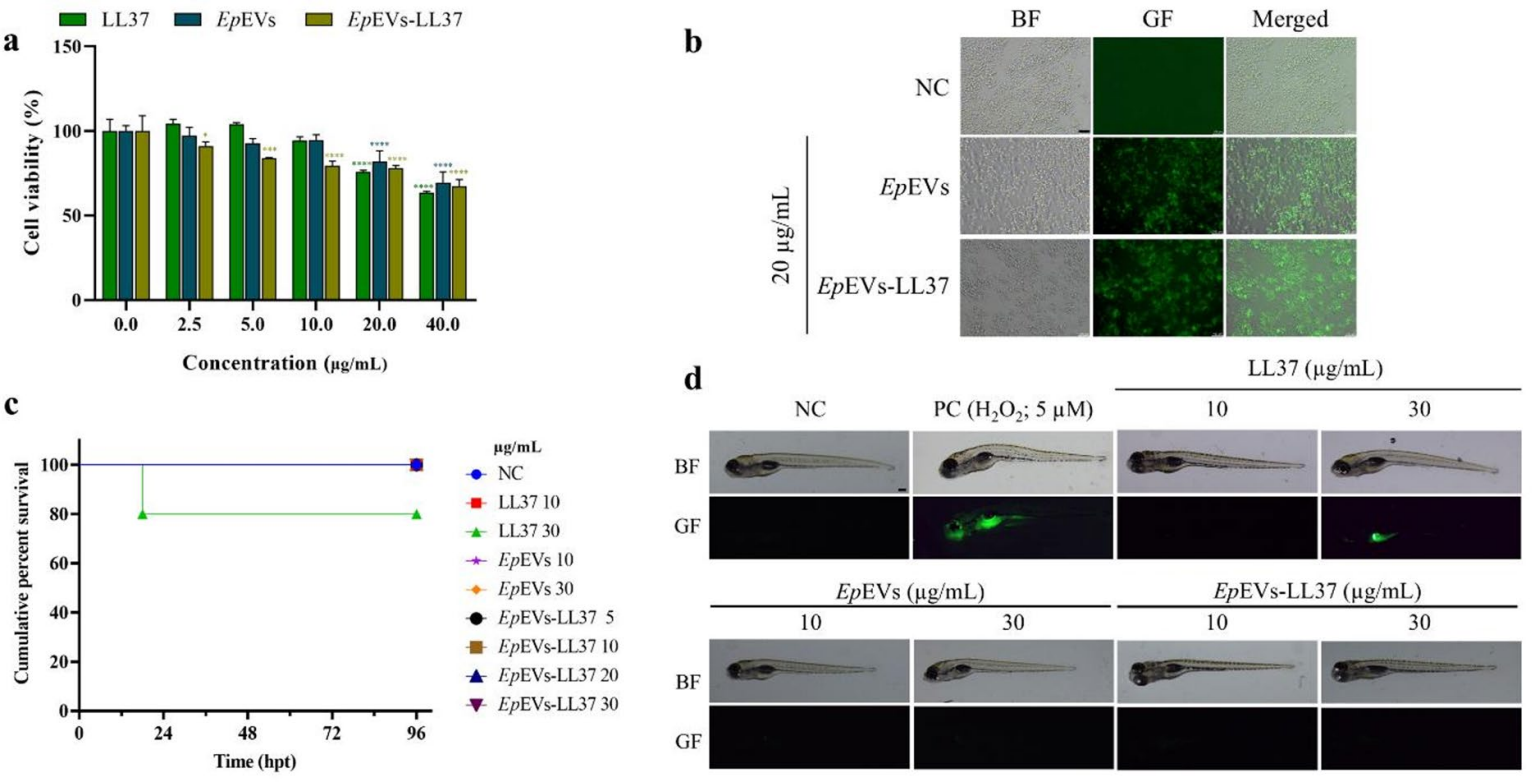
Toxicity and cellular internalization assessment of *Ep*EVs-LL37. (**a**) Cell viability of Raw 264.7 cells treated with varying concentrations of *Ep*EVs-LL37 (0–40 µg/mL) was evaluated using the Cellrix^®^ cytotoxicity assay kit. (**b**) Cellular internalization of fluorescently labeled *Ep*EVs-LL37 in Raw 264.7 cells was visualized by fluorescence microscopy. (**c**) In vivo toxicity was assessed in zebrafish larvae (60 hpf) treated with *Ep*EVs-LL37 (0–30 µg/mL), and cumulative percent survival was recorded up to 96 hpt. Supplementary Fig. S1 shows the cumulative survival rate for each treatment group. (**d**) In vivo ROS generation in zebrafish larvae at 96 hpt was determined by DCFHDA staining. H_2_O_2_ (5 µM) was used as the positive control. All experiments were performed in triplicate, and data are presented as mean ± SEM. (BF - Bright field; GF - Green fluorescence) (* = *p* < 0.05. ** = *p* < 0.01, *** = *p* < 0.001, **** = *p* < 0.0001) (Scale bar = 100 μm in **b** and 250 μm in **d**).

The cellular toxicity of *Ep*EVs-LL37 was evaluated using Raw 264.7 cells, in comparison with LL37 and *Ep*EVs alone (Fig. 2a). Both *Ep*EVs and *Ep*EVs-LL37 (20 µg/mL) showed relatively low toxicity compared to LL37. At a higher concentration of *Ep*EVs-LL37 (40 µg/mL), cell viability was decreased to 67.29%, which was slightly higher than LL37 (63.47%). According to the ISO 10993-5 standards, this range of cytotoxicity (60–80%) is considered “weakly toxic”^17^. Furthermore, internalization studies showed that *Ep*EVs-LL37 were taken up by Raw 264.7 cells with similar efficiency to that of *Ep*EVs alone, indicating that the encapsulation of LL37 did not affect the internalization efficiency (Fig. 2b). In zebrafish larvae, no mortality was observed for *Ep*EVs-LL37 or *Ep*EVs up to 96 h post-treatment (hpt) (Fig. 2c). In contrast, LL37 (30 µg/mL) resulted in a 20% reduction of the cumulative survival (%) at 96 hpt. This finding was supported by reactive oxygen species (ROS) generation analysis. Green fluorescence, indicative of ROS, was not detected in larvae treated with *Ep*EVs or *Ep*EVs-LL37, while slight green fluorescence was observed in the LL37 (30 µg/mL) treated group (Fig. 2d). However, compared to the positive control group (PC = H_2_O_2_; 5 µM), green fluorescence was markedly lower in LL37-treated larvae. Collectively, these results suggest that encapsulating LL37 in *Ep*EVs effectively reduces its toxicity at equivalent dose (0–40 µg/mL) in both Raw 264.7 cells and zebrafish larvae.

### Antibacterial activity of *Ep*EVs-LL37 against *E. piscicida*

**Fig. 3:**
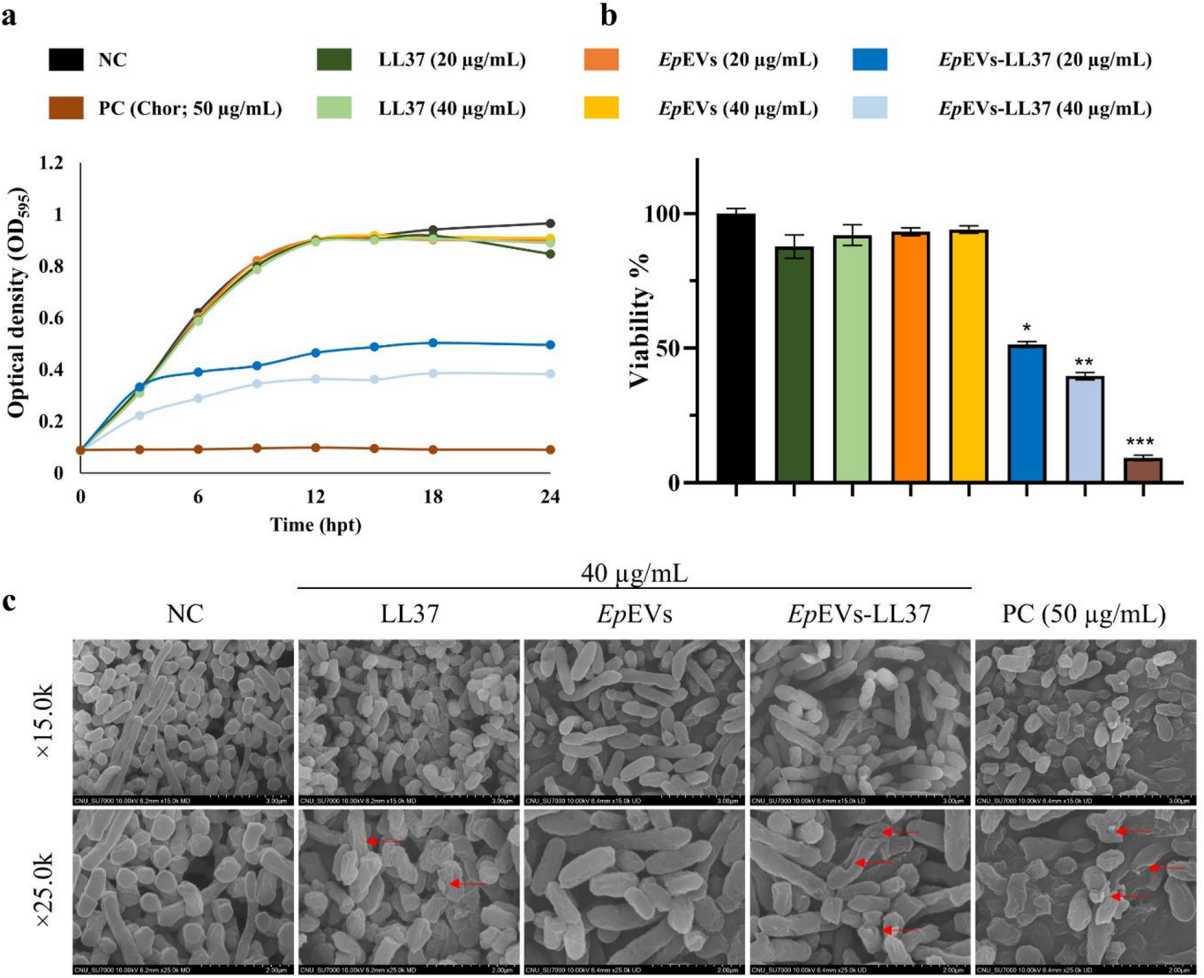
Antibacterial activity of *Ep*EVs-LL37 against *E. piscicida*. (**a**) Time-kill kinetics of *E. piscicida* treated with LL37, *Ep*EVs, or *Ep*EVs-LL37. (**b**) Cell viability of *Ep*EVs-LL37-treated *E. piscicida* was evaluated using MTT assay. (**c**) Field emission scanning electron microscopy (FE-SEM) analysis of *E. piscicida* treated with LL37, *Ep*EVs, or *Ep*EVs-LL37. Bacterial cultures of *E. piscicida* (OD_595_ ~ 0.1) were treated with LL37, *Ep*EVs, or *Ep*EVs-LL37 (40 µg/mL) for 12 h prior to fixation and dehydration. Red arrows indicate the deformation and structural damage of bacterial cells resulting from each treatment. (* = *p* < 0.05. ** = *p* < 0.01, *** = *p* < 0.001, **** = *p* < 0.0001).

The antibacterial activity of *Ep*EVs-LL37 was assessed against *E. piscicida* using time-kill kinetics with the microdilution method (Fig. 3a). At the highest concentration (40 µg/mL), *Ep*EVs-LL37 caused a slight reduction in optical density at 595 nm (OD_595_) at 3 hpt, indicating a slow antibacterial effect. By 12 hpt, NC, LL37, and *Ep*EVs alone-treated groups showed maximum bacterial growth (highest OD_595_), whereas the *Ep*EVs-LL37-treated groups showed significantly lower OD_595_ values [minimum inhibitory concentration (MIC) = 20 µg/mL]. However, complete bacterial inhibition was not achieved even at 40 µg/mL of *Ep*EVs-LL37, which was the highest available concentration. These findings indicate that LL37 alone had no antibacterial activity, whereas encapsulation within *Ep*EVs markedly enhanced its antibacterial potential. Moreover, bacterial viability was further evaluated using the 3-(4,5-dimethylthiazol-2-yl)-2,5-diphenyltetrazolium Bromide (MTT) assay (Fig. 3b). Treatment with LL37 or *Ep*EVs alone had no distinguishable reduction in bacterial viability at 24 hpt. In contrast, *Ep*EVs-LL37 treatment at 20 and 40 µg/mL significantly (*p < 0.05*) decreased bacterial viability in a dose-dependent manner compared to the negative control (NC) group, although viability reduction was less pronounced than that observed with the PC group.

To examine the morphological changes, bacterial cells were observed by field emission scanning electron microscopy (FE-SEM) at 12 hpt with LL37, *Ep*EVs, and *Ep*EVs-LL37 (Fig. 3c). Bacterial cells from the NC and *Ep*EVs-treated groups retained smooth and intact surfaces, with no obvious surface damage. The LL37-treated group showed noticeable cell shrinkage and rough surfaces compared to the NC, without major cell wall disruption. In contrast, *Ep*EVs-LL37 treatment caused pronounced morphological alterations, including cell deformation, cracks, and pores, with less shrinkage compared to the LL37-only treated group. The most severe cell damage occurred in the PC (Chloramphenicol; 50 µg/mL), which showed extensive cell deformation, shrinkage, and pore formation. Collectively, these results indicate that while LL37 alone attributed to limited cell surface alterations in *E. piscicida*, its encapsulation in *Ep*EVs effectively enhanced the antibacterial activity by promoting cell wall disruption.

### Oxidative stress and bacterial membrane permeability changes in *E. piscicida* treated with *Ep*EVs-LL37

**Fig. 4:**
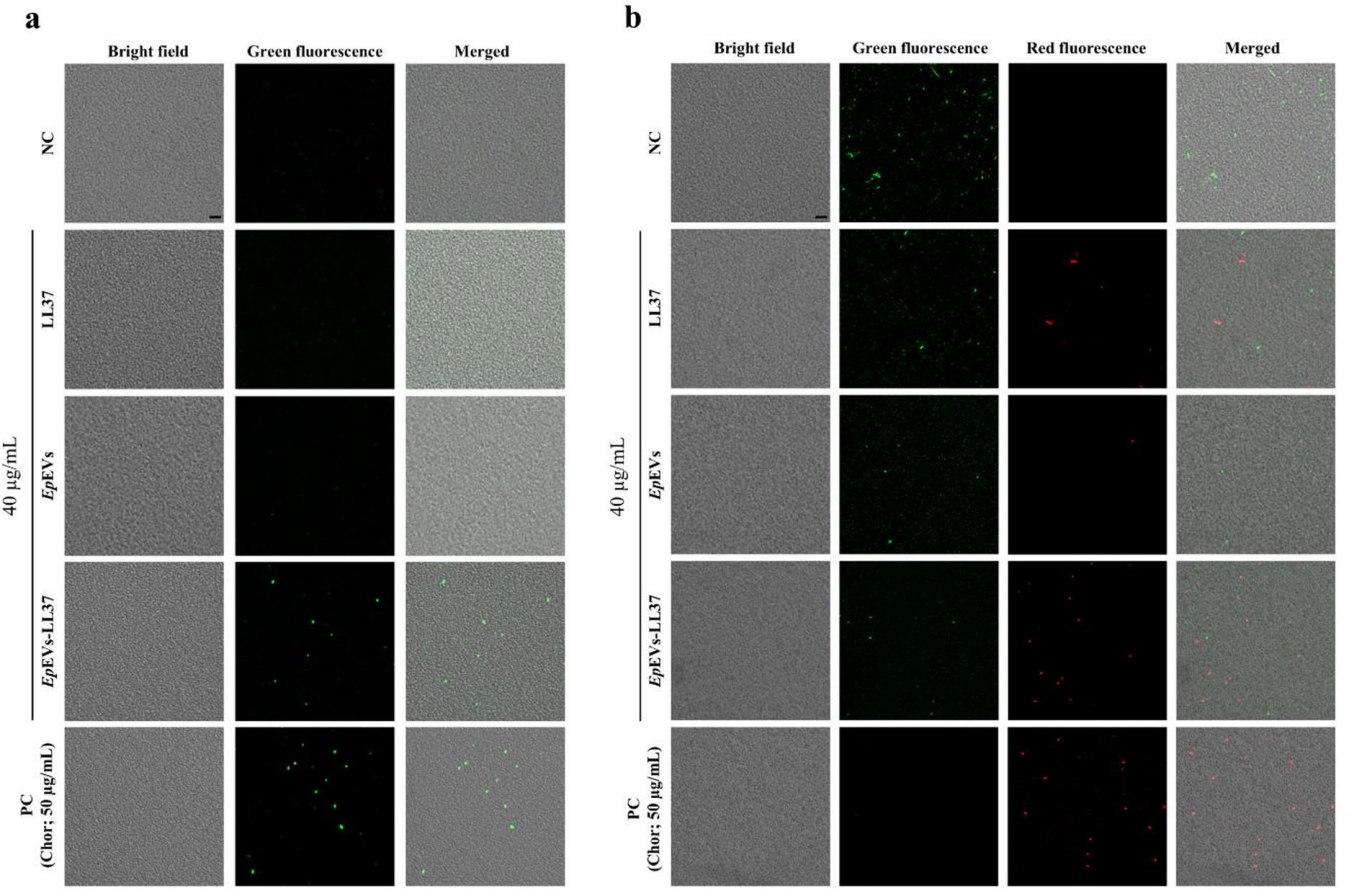
Analysis of oxidative stress and cell viability of *E. piscicida* treated with *Ep*EVs-LL37 using confocal microscopy. *E. piscicida* cells were treated with LL37, *Ep*EVs, or *Ep*EVs-LL37 for 12 h. Chloramphenicol (Chor; 50 µg/mL) was used as the positive control (PC). (**a**) ROS generation was visualized using DCFHDA staining (green fluorescence). (**b**) Changes in bacterial membrane permeability and cell death were assessed via simultaneous staining with PI (red fluorescence) and FDA (green fluorescence). (Scale bar = 5 μm).

The generation of oxidative stress in *E. piscicida* treated with LL37, *Ep*EVs, and *Ep*EVs-LL37 was assessed using 2’7’dichlorodihydro-fluorescein diacetate (DCFHDA) stained confocal microscopy (Fig. 4a). Compared with the PC, NC and *Ep*EVs-treated groups showed no detectable green fluorescence. In contrast, the LL37-only group showed a weak green fluorescence, whereas the *Ep*EVs-LL37-treated group displayed a markedly stronger green fluorescence, indicating enhanced ROS generation, implying increased oxidative stress compared to the LL37-alone group.

To further examine bacterial viability and membrane integrity, propidium iodide (PI) and fluorescein diacetate (FDA)-staining followed by confocal microscopy was conducted on the same treatment groups (Fig. 4b). PI penetrates compromised membranes and binds to DNA, producing red fluorescence in dead cells, while viable cells hydrolyze FDA to fluorescein, which emits green fluorescence^18^. The NC and *Ep*EVs-treated groups predominantly showed green fluorescence, indicating that the majority of the bacterial cells were alive. The LL37-only group exhibited a mix of green and red fluorescence, suggesting partial cell death. Notably, the *Ep*EVs-LL37-treated group showed elevated red fluorescence with minimal green fluorescence, indicating extensive bacterial cell death. These observations are consistent with the ROS generation, suggesting that encapsulation of LL37 within *Ep*EVs enhances its antibacterial activity by promoting oxidative stress and increasing membrane permeability, leading to elevated bacterial cell death.

### In vitro Immunomodulatory effect of *Ep*EVs-LL37

**Fig. 5:**
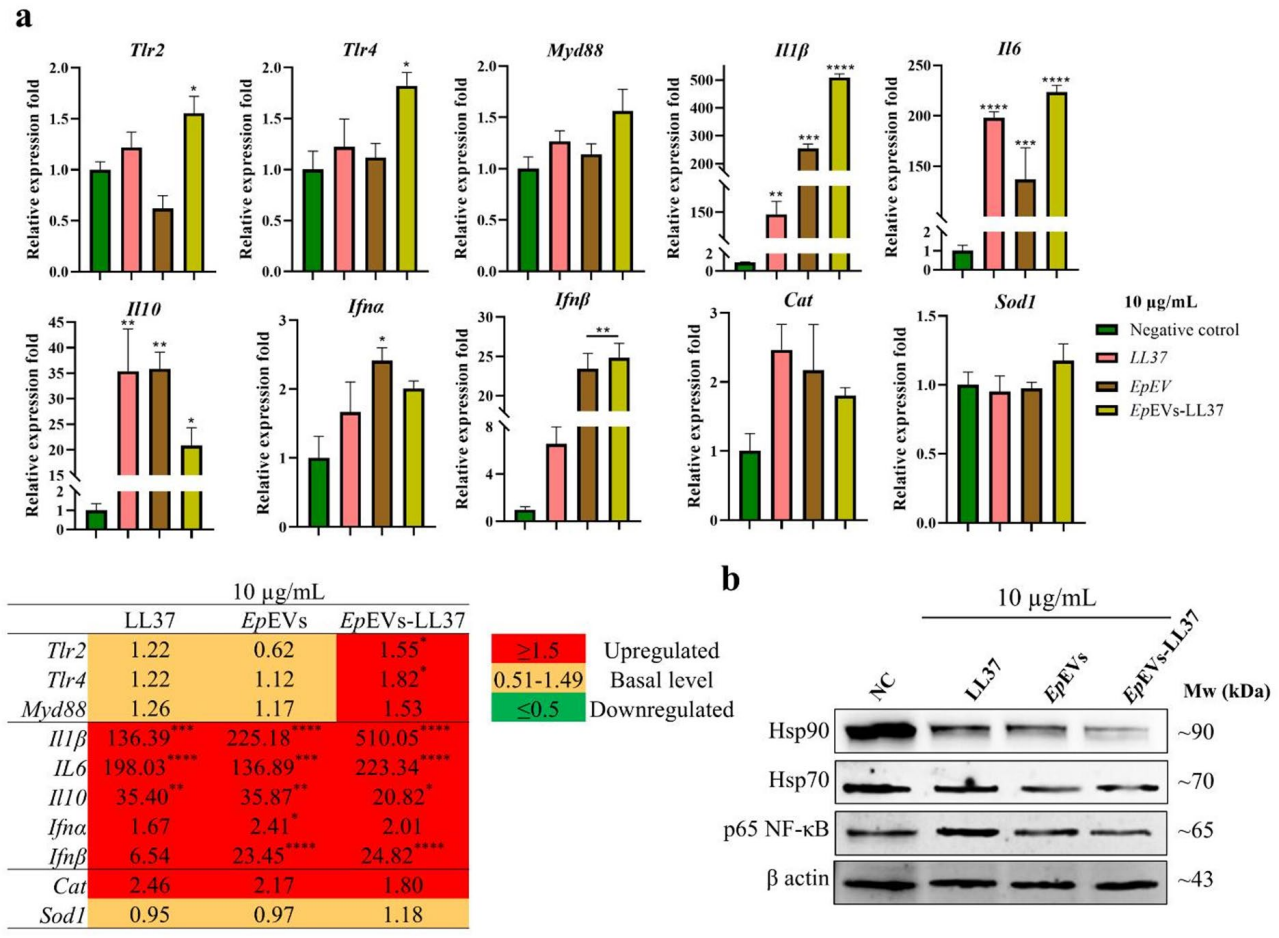
Analysis of transcriptional regulation and protein expression in *Ep*EVs-LL37-treated Raw 264.7 cells. (**a**) Gene expression analysis was performed in Raw 264.7 cells with *Ep*EVs-LL37 using qRT-PCR. *Gapdh* was used as the housekeeping gene, and β actin served as the housekeeping protein. The selected immune functional genes were analyzed by opting for the 2^−ΔΔCT^ method. (**b**) Western blot analysis was conducted to evaluate the expression of selected proteins. Cropped blots are shown in the figure, and the corresponding full blots are provided in Supplementary Fig. S2. Data are presented as mean relative expression fold compared to the PBS-treated negative control (NC) group ± SEM (* = *p* < 0.05. ** = *p* < 0.01, *** = *p* < 0.001, **** = *p* < 0.0001).

The transcriptional regulation of selected immune-related genes and proteins were analyzed to determine the immunomodulatory and antibacterial effects of *Ep*EVs-LL37 in Raw 264.7 macrophages for 24 h (Fig. 5). Pattern recognition receptors (PRRs), including toll-like receptors (*Tlr2* and *Tlr4*) were upregulated in cells treated with *Ep*EVs-LL37 (10 µg/mL) compared to the NC and *Ep*EVs-alone treatment (Fig. 5a). Furthermore, myeloid differentiation primary response 88 (*Myd88*), an upstream adaptor protein in the nuclear factor (NF)-κB signaling pathway was also upregulated. Pro-inflammatory cytokines, interleukin (*Il*)*1β* and *Il6*, were highly upregulated in all treatment groups, although *Ep*EVs-LL37-treated cells showed significantly higher fold increases (510.05-fold and 223.34-fold, respectively). Interestingly, antiviral response-related genes, interferon (*Ifn*)*α* and *Ifnβ*, were upregulated across all treatments, with *Ifnβ* showing higher expression levels than that of *Ifnα*. The antioxidative gene catalase (*Cat*) was upregulated in all treatments, while superoxide dismutase (*Sod*)1 was at a basal level.

Western blot analysis was conducted to determine the selected immune-related protein expression following *Ep*EVs-LL37 treatment (Fig. 5b). Heat shock proteins (Hsp90 and Hsp70) were highly expressed in the NC, which is a common indicator of ongoing stress. However, both *Ep*EVs and *Ep*EVs-LL37 treatments resulted in lower levels of Hsp90 and Hsp70 compared to the LL37 and NC groups, suggesting a reduced stress response. Furthermore, expression of p65 NF-κB was comparatively higher in the LL37-only treatment than in the *Ep*EVs-LL37 treatment group, implying that *Ep*EVs-LL37 may modulate NF-κB signaling more effectively, leading to a balanced immune activation.

### In vivo Immunomodulatory effect of *Ep*EVs-LL37

**Fig. 6:**
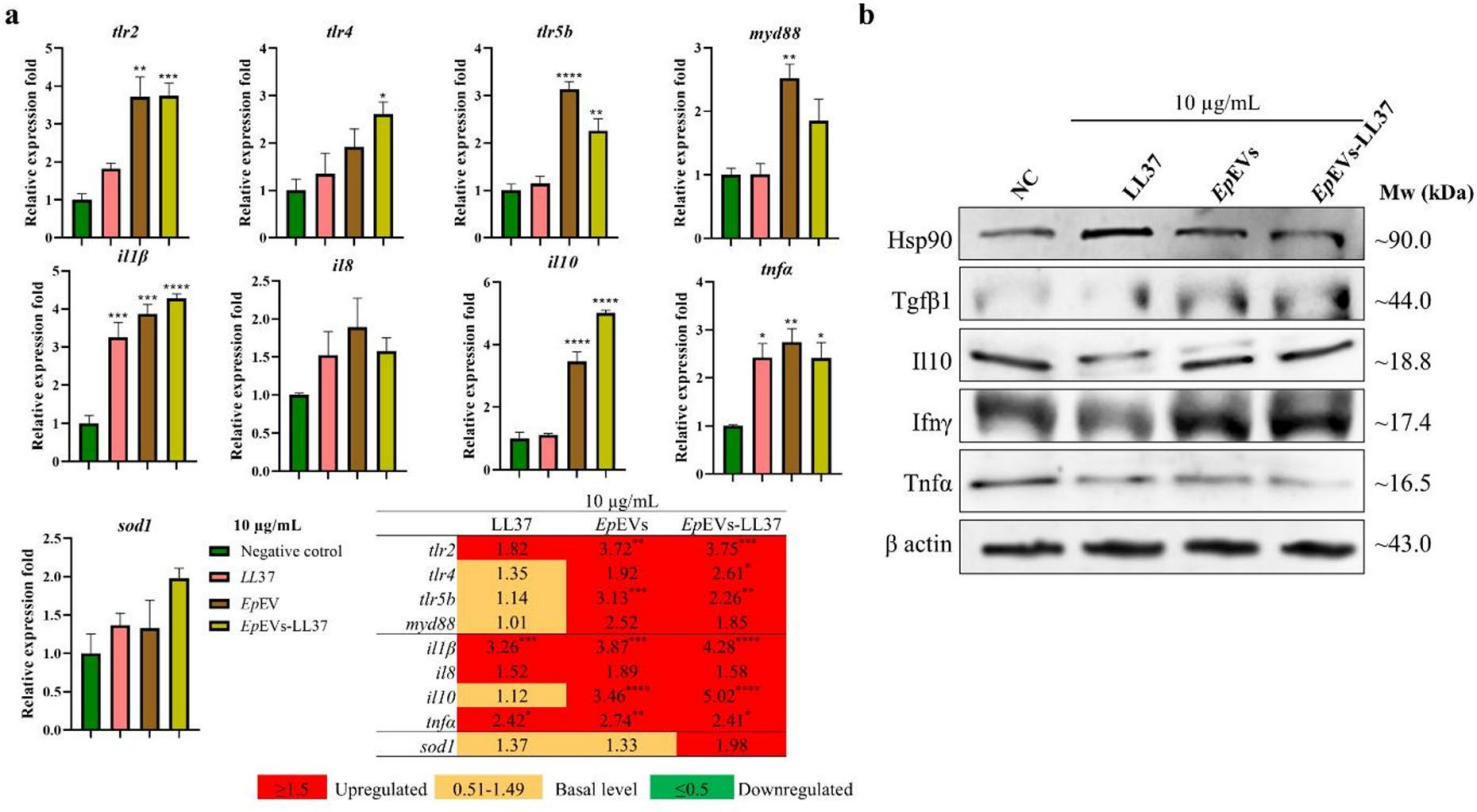
In vivo immunomodulatory effect of *Ep*EVs-LL37. (**a**) Transcriptional regulation and (**b**) protein expression analysis of *Ep*EVs-LL37-treated zebrafish larvae. Zebrafish larvae (60 hpt) were pre-treated with LL37, *Ep*EVs, or *Ep*EVs-LL37 (10 µg/mL). Relative fold expression of selected genes was quantified using the 2^−ΔΔCT^ method, with *β actin* serving as the housekeeping gene for normalization. For the Western blot analysis, 25 µg of crude protein from zebrafish larval lysate was loaded per well and electrophoresed. Cropped blots are shown in the figure, and the corresponding full blots are provided in Supplementary Fig. S3. Data are presented as mean relative fold compared to the PBS-treated negative control (NC) group ± SEM (* = *p* < 0.05. ** = *p* < 0.01, *** = *p* < 0.001, **** = *p* < 0.0001).

In vivo immunomodulatory effects of *Ep*EVs-LL37 were evaluated in zebrafish larvae at 24 hpt by assessing the expression of selected immune-related genes (Fig. 6a). Pattern recognition receptor genes (*tlr2*, *tlr4*, and *tlr5b*) were highly upregulated in both *Ep*EVs and *Ep*EVs-LL37 treatment groups. In contrast, only *tlr2* was upregulated (1.82-fold) in the LL37-treated group. The expression of *myd88* remained at a basal level in the LL37-treated larvae, while upregulation was observed in both *Ep*EVs and *Ep*EVs-LL37 groups. Pro-inflammatory genes (*il1β*,* il8*, and *tnfα*) were upregulated both in LL37 and *Ep*EVs-LL37-treated larvae. Conversely, the anti-inflammatory gene *il10* showed the highest expression (5.02-fold) in the *Ep*EVs-LL37 group, whereas the LL37-treated group remained at a basal level (1.12-fold). Antioxidative gene *sod1* was also upregulated following *Ep*EVs-LL37 treatment, while both LL37 and *Ep*EVs treatments maintained basal expression levels.

Protein-level expression of *Ep*EVs-LL37 using Western blotting further supported these findings (Fig. 6b). The stress response protein Hsp90 was elevated in the LL37-treated group compared to the NC. However, its expression was comparatively lower in both *Ep*EVs and *Ep*EVs-LL37 treatments, indicating reduced cellular stress. The Ifnγ level was increased in the *Ep*EVs-LL37-treated group compared to the NC, suggesting immune activation. In contrast, Il10 protein levels showed similar expressions across all treatment groups except LL37, further supporting an enhanced anti-inflammatory response with *Ep*EVs-LL37. Additionally, Tnfα levels were decreased following *Ep*EVs-LL37 treatment, further providing evidence of its anti-inflammatory and immunoregulatory potential.

### In vitro wound-healing effect of *Ep*EVs-LL37

**Fig. 7:**
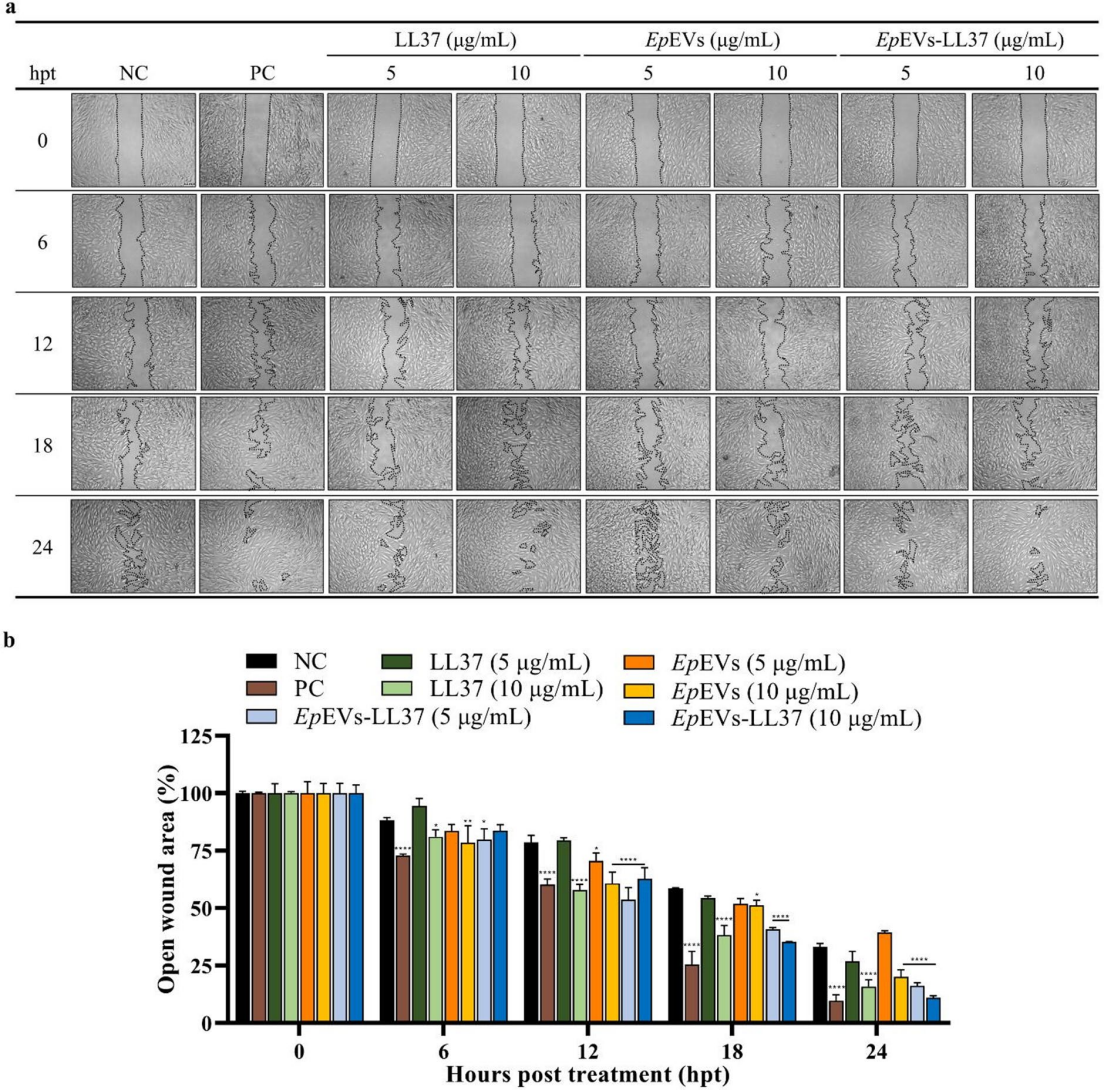
In vitro wound-healing activity of *Ep*EVs-LL37 evaluated using a cell migration assay. LL37, *Ep*EVs, or *Ep*EVs-LL37 (5 and 10 µg/mL) were treated to HDF cells and observed the cell migration. (**a**) Representative light microscope images showing the cell-free wound area at different time points. (**b**) Quantification of wound area (%) was performed using ImageJ software. The open wound area (%) was plotted against hours post-treatment (hpt). Data were compared with the negative control (NC) group at each time point and reported as means ± SEM. Statistical analysis was performed using two-way ANOVA followed by Dunnett’s multiple comparison test. (Scale bar = 100 μm; * = *p* < 0.05. ** = *p* < 0.01, *** = *p* < 0.001, **** = *p* < 0.0001).

A cell migration assay was performed using human dermal fibroblast (HDF) cells to evaluate the in vitro wound-healing efficacy of *Ep*EVs-LL37. Fetal bovine serum (FBS)-supplemented media was used as the PC, which showed a drastic reduction in the open wound area at all time points compared to other treatments (Fig. 7a). Quantitative image analysis revealed that at 6 hpt except for LL37 (5 µg/mL), all other treatment groups showed a higher rate of wound closure (Fig. 7b). However, no visible difference was observed between *Ep*EVs and *Ep*EVs-LL37 at this time point. A distinct difference among treatments was observed after 18 hpt, where LL37 (10 µg/mL) and *Ep*EVs-LL37 (5 and 10 µg/mL) showed rapid wound closure compared to *Ep*EVs. Apart from the PC, the lowest wound area (%) was recorded for *Ep*EVs-LL37 (10 µg/mL) at 24 hpt (11.01 ± 0.81%), which was only slightly higher than that of the PC group (9.61 ± 2.71%). A clear dose-dependent enhancement in wound-healing was observed for *Ep*EVs-LL37 after 18 hpt. Collectively, these results indicate that *Ep*EVs-LL37 process a prominent wound-healing capability in HDFs compared to LL37 alone.

### In vivo wound-healing effect of *Ep*EVs-LL37

**Fig. 8:**
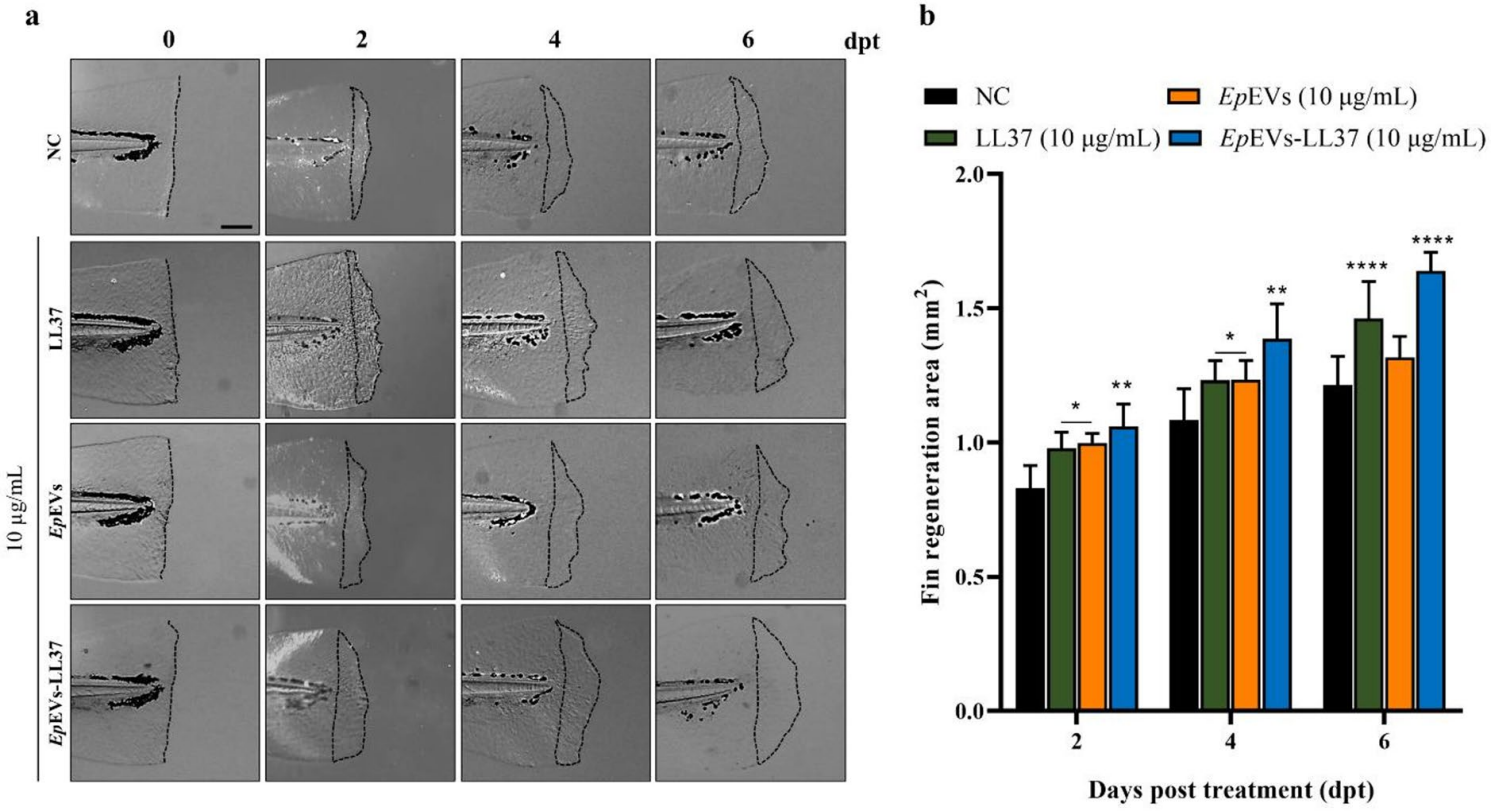
In vivo wound-healing activity of *Ep*EVs-LL37 in zebrafish larvae. LL37, *Ep*EVs, or *Ep*EVs-LL37 (5 and 10 µg/mL) were treated to zebrafish larvae (60 hpf) after caudal fin amputation. (**a**) Representative images of regenerating fins were captured at 0, 2, 4, and 6 dpt under a light microscope at each time point. (**b**) Quantification of regenerated fin area (%) was performed using ImageJ software, and the results were plotted against days post-treatment (dpt). Data were compared with the negative control (NC) group at each time point and reported as means ± SEM. Statistical significance was determined using two-way ANOVA followed by Dunnett’s multiple comparison test. (Scale bar = 250 μm; * = *p* < 0.05, ** = *p* < 0.01, *** = *p* < 0.001, **** = *p* < 0.0001).

To further assess the wound-healing potential in *Ep*EVs-LL37, an in vivo fin regeneration assay was conducted using 60 h post-fertilization (hpf) zebrafish larvae. According to Fig. 8a, *Ep*EVs-LL37-treated larvae showed accelerated fin regeneration compared to other treatment groups, where the NC group displayed the least fin regeneration. Quantitative analysis of the regenerated fin area demonstrated a significant increase (*p < 0.05*) in the *Ep*EVs-LL37-treated group (1.21 ± 0.11 mm^2^) at 2 days post-treatment (dpt), compared to the NC group (0.83 ± 0.08 mm^2^) (Fig. 8b). Notably, both LL37 and *Ep*EVs alone showed similar wound-healing activity at 2 and 4 dpt. However, by 6 dpt, a significant (*p < 0.05*) enhancement in fin regeneration activity was observed for both LL37 (1.32 ± 0.08 mm^2^) and *Ep*EVs-LL37 (1.64 ± 0.07 mm^2^), although *Ep*EVs-LL37 demonstrated the highest wound-healing capacity throughout the experiment period. The NC consistently exhibited the lowest fin regeneration at all time points.

## Discussion

BEVs have been recognized as promising drug-delivery agents capable of targeting various tissues, including the brain, gut, and bones, and have demonstrated therapeutic potential against diseases^4,19^. According to Liu et al., BEVs are currently being utilized in cancer therapy and tissue regeneration owing to their inherent physicochemical properties and immunomodulatory functions^5^. Although several naïve BEVs have been utilized for therapeutic applications, engineering BEVs, specifically through the incorporation of protein cargo, portray a valuable yet unexplored strategy for targeted drug delivery. Therefore, we extended our previous research by encapsulating LL37 within *Ep*EVs and evaluated its anti-bacterial activity against *E. piscicida*. In parallel, we assessed the immunomodulatory and wound-healing properties of *Ep*EVs-LL37 in Raw 264.7 cells and zebrafish models.

Since BEVs are derived from living bacterial cells, they retain many of the molecular components of their parent organisms. Their composition typically includes lipids, proteins (membrane-bound and cytoplasmic), nucleic acids (DNA and RNA), and LPS-like toxins^20^. These bacterial constituents form the foundation of BEVs-mediated immunomodulation, as BEVs that enter host cells are often misidentified as intact bacteria rather than non-living vesicular nanoparticles. As a result, BEVs can stimulate immune activation and enhance host immunoprotection without the infection^5^. Additionally, BEVs play a crucial role in bacterial communication. They can be rapidly internalized by bacteria of the same or different species with minimal resistance^21^. Therefore, engineered BEVs loaded with AMPs can penetrate bacteria that have developed resistance to those AMPs, thereby reducing the AMP resistance and improving disease control^21^. Based on that, we co-incubated the LL37 AMP with *Ep*EVs and produced the *Ep*EVs-LL37 and subsequently evaluated their therapeutic potential against *E. piscicida* infection.

LL37 is a cationic (+ 6) AMP composed of 37 amino acids and exhibits a characteristic α-helical conformation^22^. It represents the active form of the only human cathelicidin type AMP, and has been reported to possess a broad spectrum of antiviral (e.g., Influenza A), antifungal (*Candida auris*), and antibacterial (*Streptococcus aureus*) activities^23–25^. In addition to its antimicrobial properties, LL37 demonstrated multiple biological functions, including wound-healing^26^, anti-inflammatory and immunomodulatory activity via Tlr regulation^27^, and immune-adjuvant effects such as stimulation of angiogenesis^28^, and enhanced ischemic hind leg collateral circulation in mice^29^. Clinical studies have demonstrated that LL37 is non-toxic when applied topically to patients with venous leg ulcers and can promote wound-healing^30^. The principal mechanism of action of LL37 involves membrane disruption, attributed to the cationic nature of the peptide. In addition, LL37 can modulate bacterial immune responses, leading to indirect antibacterial effects^27^. Neshani et al. reported the antibacterial activity of LL37 against a diverse array of human pathogenic bacteria, including Gram-positive Actinomyces, Streptococcus, Bacillus, Clostridium, Listeria, and Peptostreptococcus and Gram-negative Pseudomonas, Escherichia, Salmonella, Klebsiella, Vibrio, Neisseria, Haemophilus, Porphyromonas, Helicobacter, Fusobacterium, and Stenotrophomonas genera^15^. However, to date, no published studies have reported the activity of LL37 against the fish pathogenic *E. piscicida*.

Different strategies have been used to load cargo into EVs, including electroporation, sonication, co-incubation, freeze-thaw cycle, fusion, dialysis, and extrusion^7^. Among these, the co-incubation method is not only convenient but also enables passive loading of AMPs into the BEVs, preserving their vesicular structure while minimizing drug degradation caused by external physical or chemical loading forces^6^. Interestingly, compared with the *Ep*EVs reported in our previous study, both F1 and F2 formulations exhibited no significant difference in NTA, TEM, or zeta potential measurements, confirming that the co-incubation method maintained the physicochemical characteristics of *Ep*EVs after loading with LL37^16^. Although co-incubation generally results in a lower EE, as shown in Table 1, this limitation was mitigated by increasing the initial amount of LL37, which nearly doubled the EE of F2 (61.67%) compared with F1 (32.67%). In contrast, Huang et al. reported variations in particle size, zeta potential, and even protein content when different antibiotics were loaded into *Acinetobacter baumannii*-derived EVs, suggesting that the physicochemical properties may depend on the molecular weight or nature of the cargo^8^. Based on these results, the F2 formulation was selected as *Ep*EVs-LL37 for further studies. To assess the stability of *Ep*EVs-LL37 under gastrointestinal-like conditions, pepsin was used ex vivo to mimic the acidic environment of the stomach^31^. Pepsin is known to digest surface proteins such as mucin 5B and CD9 in salival EVs^31^. Thus, the observed increase in mean particle size may result from surface protein modifications or internalization of digested peptides. However, the observed increase in zeta potential after pepsin treatment under acidic conditions did not coincide with the results of Zang et al., who reported a zeta potential of + 0.28 mV at low pH for edible ginger-derived nanoparticles^32^. Furthermore, Midekessa et al. demonstrated that the negative surface charge of EVs increases with rising pH^33^. Therefore, this phenomenon warrants further investigation, particularly focusing on the membrane charge dynamics of EVs under gastrointestinal conditions to better evaluate their stability.

During safe dose determination, the viability of Raw 264.7 cells treated with LL37 (20 µg/mL) was 75.85 ± 0.46%, indicating low toxicity. According to Aidoukovitch et al., synthetic LL37 reduced cell viability of human monocytic cells THP-1 by approximately 20% at 9 µM (40.5 µg/mL) after 4 hpt, which suggests a higher level of toxicity compared with our findings^24^. Similarly, Habibi et al. reported that LL37-conjugated iron oxide nanoparticles induced cancer cell death through elevated oxidative stress^34^. In general, oxidative stress results from the excessive accumulation of ROS in living organisms^35^. This may explain the relatively higher ROS production observed in LL37-treated zebrafish larvae with increased mortality. Furthermore, to determine whether LL37 encapsulation affected cellular internalization dynamics, *Ep*EVs-LL37 were fluorescently labeled and compared with labeled *Ep*EVs. Both treatments showed similar green fluorescent intensities, indicating comparable levels of cellular internalization. These results suggest that encapsulating LL37 within *Ep*EVs effectively reduced its apparent cytotoxicity in both cells and zebrafish larvae while maintaining efficient cellular uptake.

Although LL37 exhibits broad-spectrum activity against various Gram-negative and Gram-positive bacteria, the time-kill kinetics and MTT assay results revealed that LL37 had no detectable antibacterial effect against *E. piscicida*. Joo et al. reported that certain Gram-negative bacteria possess adaptive mechanisms that interfere with AMP internalization, including the secretion of extracellular proteins that facilitate AMP degradation, surface modifications, cytoplasmic membrane alterations, and the use of efflux pumps to expel AMPs from the cells^36^. Similarly, Bae et al. demonstrated that bacteria can modify surface LPS to increase resistance to cationic AMPs, altering host recognition of LPS, and thereby promoting bacterial survival^37^. In particular, the O-antigen component of LPS is known to create steric hindrance, thereby impeding the approach of cationic AMPs. Moreover, acylation of lipid A facilitates the rigidity of the outer membrane, thereby reducing membrane permeability^36^. Furthermore, Zhang et al. have identified that the arnB gene mediates the development of *E. piscicida* resistance to cationic AMPs^38^. These mechanisms may explain why LL37 alone had no antibacterial effect against *E. piscicida.* Since *Ep*EVs and *Ep*EVs-LL37 share similar membrane structures and physicochemical properties, both can be internalized by *E. piscicida* without any resistance. However, *Ep*EVs-LL37 effectively induced bacterial death and membrane disruption by LL37 within *Ep*EVs. This was further confirmed by FE-SEM observations, where *Ep*EVs-LL37-treated cells displayed visible membrane damage and morphological alterations, similar to those observed in the PC (Chloramphenicol; 50 µg/mL), while LL37, *Ep*EVs alone caused no noticeable membrane damage. Similarly, confocal microscopy of PI/FDA-stained samples revealed that *Ep*EVs-LL37 altered *E. piscicida* membrane permeability, increasing the ratio of dead to live bacterial cells. These findings are consistent with the results from the time-kill kinetics and MTT assays. Uncontrolled accumulation of ROS in bacterial cells can induce oxidative stress and macromolecular damage to protein, DNA, and lipids, leading to eventual cell death^18^. This explains the higher ROS levels observed in *Ep*EVs-LL37-treated cells compared with those treated with LL37 alone, further supporting our previous finding that *Ep*EVs-LL37 increased bacterial membrane permeability, thereby reducing cell viability and promoting cell death. Similarly, Ibrahim et al. reported that EVs derived from LPS-induced hepatocellular carcinoma cells (iEVs) coated with AMP-A (PC-iEV) exhibited enhanced antibacterial activity against *Escherichia coli*, showing a four-fold reduction in minimum bactericidal concentration (MBC) compared with AMP-A, suggesting the potential of modifying EVs for use as AMP delivery systems^39^.

Immune cells release LL37 upon activation by damage-associated molecular patterns (DAMPs) and pathogen-associated molecular patterns (PAMPs)^27^. The active form of LL37 also plays a crucial role in modulating macrophage activity. PRRs such as Tlrs recognize bacterial components, including LPS, peptidoglycan, and flagellin, thereby alerting immune cells to infection^16^. Although in vitro activation of *Tlr2* and *Tlr4* was observed for *Ep*EVs-LL37 at 24 hpt, in vivo results demonstrated activating *tlr2*, *tlr4*, and *tlr5b*, suggesting the immunostimulatory effect of *Ep*EVs-LL37. Importantly, LL37 and *Ep*EVs alone did not upregulate *Tlr2* and *Tlr4* expression, possibly due to gradual intracellular release of LL37 following internalization into Raw 264.7 cells. However, this pattern was not consistently observed in vivo. Activation of host PRRs trigger various transcriptional factors, which subsequently stimulate immune cells to release pro-inflammatory (*Il1β*,* Il6*,* Il8*, and *Tnfα*) and anti-inflammatory cytokine *Il10*^40^. These cytokines further enhance immune cell activation and prime the immune system to respond more effectively. In addition, they promote the production of type 1 Ifns, antimicrobial peptides, and chemokines, which play key roles in limiting infection and strengthening host defenses^40,41^. Consistent with this, our in vitro results demonstrated that LL37, *Ep*EVs, and *Ep*EVs-LL37 upregulated the expression of pro-inflammatory cytokines (*Il1β*,* Il6*, and *Tnfα*) and anti-inflammatory (*Il10*) cytokines as well as *Ifnα* and *Ifnβ* at varying fold-change levels. Similarly, in vivo gene expression analysis revealed that *Ep*EVs-LL37 induced upregulation of both pro-inflammatory and anti-inflammatory cytokines. Yang et al. reported that in macrophages, LL37 can both upregulate and downregulate the expression of pro-inflammatory cytokines (Il6, Il8, Tnfα) and anti-inflammatory Il10^27^. Moreover, the immunomodulatory effect of LL37 strongly depends on cell type, nature of stimulus, and peptide concentration. Tabarsa et al. reported that LL37 levels in hidradenitis suppurativa, an inflammatory skin condition, positively correlates with immune cell infiltration and the expression of pro-inflammatory cytokines (Ifnγ, Il1β, Il17, Il23, Il32, and Tnfα), which coordinate and sustain inflammation^42^. Similarly, activation of host PRR can stimulate the NF-κB signaling pathway, leading to the release of pro-inflammatory cytokines and chemokines^42^. Consistent with these findings, our results demonstrated that *Ep*EVs-LL37 treatment at 24 hpt upregulated the expression of *Myd88* in both in vitro and in vivo, as well as p65 NF-κB protein expression in Raw 264.7 cells. Although upregulation of pro-inflammatory genes and protein levels was observed in *Ep*EVs-LL37-treated zebrafish larvae and Raw 264.7 cells, the expression of stress-related proteins (Hsp70 in vitro and Hsp70 and Hsp90 in vivo) remained comparatively low, suggesting that the encapsulated product exhibits minimum toxicity^43^. Furthermore, our previous study reported that *Ep*EVs alone had similar upregulation of both pro- and anti-inflammatory cytokines in Raw 264.7 cells as well as zebrafish kidney samples^16^. Taken together, these findings indicate that when treating *Ep*EVs-LL37, the combined or cumulative effect of LL37 and *Ep*EVs should be considered.

Although the primary function of AMPs is to combat infection caused by pathogenic microorganisms and enhance host defense, recent studies have highlighted additional roles of AMPs in modulating inflammation and wound-healing^44^. Consistently, the wound-healing activity of LL37 has been reported in multiple publications^14,45,46^. Therefore, a comparative wound-healing analysis was conducted both in vitro (cell migration assay) and in vivo (zebrafish larval fin regeneration assay) to evaluate whether the encapsulation process altered its wound-healing activities. The HDF cell migration assay revealed that at 24 hpt, *Ep*EVs-LL37 exhibited slightly higher wound closure (%) compared to LL37 alone. Furthermore, BEVs can carry proteins that contribute to wound repair. For example, Flagellin, a common virulence-associated protein of Gram-negative bacteria, has been reported to possess anti-tumor and radioprotective activities by regulating radiation-induced tissue damage^47^. Moreover, Gao et al. reported that *Pseudomonas aeruginosa*-derived flagellin stimulated porcine corneal epithelial cells, enhancing production of AMPs, cell migration, proliferation, and wound-healing activity^48^. Similarly, the GroEL protein (also known as the Hsp60), a molecular chaperone, has been shown to promote cell proliferation and fin regeneration in zebrafish larvae as well as wound-healing activity in diabetic mice^49^. Proteomic analysis of *Ep*EVs confirmed the presence of both flagellin and chaperonin GroEL, which may contribute synergistically to the enhanced wound-healing effect of *Ep*EVs-LL37^16^. Furthermore, this augmented regenerative potential was validated in the in vivo fin regeneration assay, demonstrating that *Ep*EVs-LL37 possessed superior wound-healing and tissue regenerative properties compared to using LL37 alone.

## Conclusion

Taken together, we successfully optimized and encapsulated LL37 into *Ep*EVs using a passive co-incubation method and characterized their physicochemical and morphological properties. Although direct treatment with LL37 showed no antibacterial effect against *E. piscicida*, *Ep*EVs-LL37 exhibited markedly enhanced antibacterial activity by reducing bacterial membrane resistance to LL37. Encapsulation of LL37 within *Ep*EVs effectively masked the peptide, facilitating higher bacterial uptake, which led to production of elevated ROS levels, increased membrane permeability, and subsequent bacterial cell death. Moreover, *Ep*EVs-LL37 demonstrated comparatively lower cytotoxicity towards Raw 264.7 cells and zebrafish larvae, while maintaining cellular internalization similar to that of naïve *Ep*EVs. Both in vitro and in vivo assays revealed pronounced immunomodulatory activity following *Ep*EVs-LL37 treatment. In addition, *Ep*EVs-LL37 significantly enhanced wound-healing capabilities, as evidenced by accelerated cell migration and fin regeneration in HDF cells and zebrafish larvae, likely due to the synergistic effects of both LL37 and *Ep*EVs. Future studies should focus on elucidating the detailed molecular mechanisms underlying the enhanced antibacterial and immunomodulatory properties of *Ep*EVs-LL37. Furthermore, exploring their therapeutic potential in infection models and evaluating their in vivo antibacterial-associated wound-healing efficacy would advance the development of *Ep*EVs-LL37 as a therapeutic candidate against *E. piscicida* infection in aquaculture.

## Materials and methods

### *Ep*EVs isolation and encapsulation of LL37

Isolation and characterization of *Ep*EVs were conducted as described in our previous publication^16^. Protein concentration of *Ep*EVs was quantified using Bradford’s assay. Encapsulation of LL37 into *Ep*EVs was performed by the co-incubation method. Different amounts of LL37 (0.2 and 0.4 mg) were used with the same amount of *Ep*EVs (0.2 mg) for the ratio optimization strategy to obtain the highest EE%. Briefly, LL37 (stock solution: 0.4 mg/mL) and *Ep*EVs were mixed (following the ratios mentioned in Table 2) in ultracentrifuge tubes (Eppendorf Himac Technologies Co. Ltd., Tokyo, Japan) and incubated at 30 °C for 3 h. After incubation, ultracentrifugation was performed at 100,000 × g for 2 h. To obtain *Ep*EVs-LL37, the supernatant was carefully separated, and the resulting pellet was dissolved in 200 µL of 0.1 μm filtered phosphate-buffered saline (PBS). The remaining LL37 (non-encapsulated) was quantified using a NanoDrop One spectrometer (Thermo Fisher Scientific, Madison, WI, USA). The EE% was calculated based on the following formula, and the isolated *Ep*EVs-LL37 were stored at -80 °C until further use.

**Table 2:** Preparation criteria of *Ep*EVs-LL37.

Description	Formulation 1 (F1)	Formulation 2 (F2)
Ratio BEVs: AMPs	1:1	1:2
Final reaction mixture volume (mL)	4	4
*Ep*EVs (0.1 mg/mL) amount (mg)	0.2	0.2
*Ep*EVs volume (mL)	2	2
Final concentration of *Ep*EVs (mg/mL)	0.05	0.05
LL37 (stock: 0.4 mg/mL) amount (mg)	0.2	0.4
LL37 volume (mL)	0.5	1
Final concentration of LL37 (mg/mL)	0.05	0.1
PBS volume (mL)	1.5	1

*AMP (LL37)*: antimicrobial peptide, *BEVs (EpEVs)*: bacterial extracellular vesicles.

$$\mathrm{EE}\%=\frac{\text{[Total quantity of LL37}-\text{Remaining quantity of LL37 in the supernatant (g)]}}{\text{Total quantity of LL37 (g)}}\times \mathrm{100}$$

Based on the highest values observed for the EE%, the optimum ratio between LL37 and *Ep*EVs was selected for further experiments. Treatment dose of *Ep*EVs-LL37 were determined based on the LL37 amount encapsulated in *Ep*EVs, back-calculated with the EE%.

### Physicochemical characteristics and stability of *Ep*EVs-LL37

Characterization of the isolated *Ep*EVs-LL37 was compared to determine whether there were significant changes with each formulation, following the methods described in our previous publication^16^. NTA was conducted using a NanoSight NS300 system (Malvern Technologies, Malvern, UK) to determine the particle size distribution and concentration. The membrane charge of *Ep*EVs-LL37 was analyzed by employing a zeta potential analyzer (Zetasizer Nano ZSP, Malvern, UK). Finally, the ultrastructural morphology was observed using FE-TEM with a Tecnai™ G2 F30 super-twin; FEI system (Hillsboro, OR, USA).

To determine the stability in acidic stomach conditions, *Ep*EVs-LL37 were treated with pepsin (0, 0.4, 2 mg/mL) (Sigma-Aldrich, St. Louis, MO, USA) following a previous publication with minor modifications^31^. *Ep*EVs-LL37 were treated with pepsin (pH 2) under different conditions and incubated at 37 °C for 3 h. After the incubation, pH was neutralized using NaOH (0.2 N; Sigma-Aldrich, St. Louis, MO, USA) solution. Treated samples were used for NTA, zeta potential, and FE-TEM analysis to evaluate their stability.

### Toxicity and cellular internalization of *Ep*EVs-LL37

The toxicity of *Ep*EVs-LL37 was evaluated in vitro and in vivo using Raw 264.7 cells and zebrafish larvae, respectively. Raw 264.7 cells were kindly provided by the Microbiology Laboratory, College of Veterinary Medicine, Chungnam National University, and wild-type (WT) zebrafish were obtained from the Zebrafish Center for Disease Modeling (ZCDM), Daejeon, Rep. of Korea. Adult zebrafish were bred according to established protocols to obtain larvae used in the study. Raw 264.7 cells were maintained and periodically subcultured in Dulbecco’s Modified Eagle Medium (DMEM) (Wellgene Co., Ltd., Gyeongsan, Rep. of Korea) supplemented with 10% FBS and 1% antibiotic mixture (Penicillin/Streptomycin) (Gibco, Grand Island, NY, USA)^16^. Cytotoxicity of *Ep*EVs-LL37 (0–40 µg/mL) was evaluated using the Cellrix^®^ Viability Assay Kit (MediFab, Geumcheon, Rep. of Korea) following the manufacturer’s instructions. To determine the in vivo toxicity, 60 hpf larvae were selected and individually treated with *Ep*EVs-LL37 (0–30 µg/mL) following the procedure described in our previous publication^16^. Cumulative percentile survival was analyzed regularly until 96 hpt. At 96 hpt, five larvae from each treatment were stained with DCFHDA (Sigma-Aldrich, St. Louis, MO, USA) (5 µg/mL). Excess stain was rinsed off using PBS, and observed under a light microscope (Nikon SMZ100, Tokyo, Japan) equipped with an SFA fluorescent filter (NIGHTSEA, Hatfield, PA, USA) to visualize the ROS generation. All zebrafish experiments were approved by the Animal Ethics Committee of Chungnam National University and conducted in accordance with its guidelines (202310 A-CNU-189). Reporting zebrafish-related experiments was done according to the guidelines provided by the Animal Research: Reporting of In Vivo Experiments (ARRIVE).

Cellular internalization of *Ep*EVs-LL37 was evaluated in comparison with *Ep*EVs after simultaneous fluorescent labeling of *Ep*EVs-LL37 lipid membrane using the ExoSparkler kit (Dojindo Molecular Technologies Inc., Rockville, MD, USA) according to the manufacturer’s recommendations. Labeled *Ep*EVs-LL37 were treated to pre-seeded Raw 264.7 cells, and cellular internalization was observed following the procedure described in our previous publication^16^.

### Antibacterial activity of *Ep*EVs-LL37 against *E. piscicida*

The antibacterial activity of *Ep*EVs-LL37 was evaluated by performing time-kill kinetics and bacterial cell viability assays. *E. piscicida* was grown in Brain Heart Infusion (BHI) plates and broth supplemented with 1% NaCl as described in our previous work^16^. Microdilution was performed in accordance with Clinical and Laboratory Standards Institute (CLSI) guidelines (M07-A10) to determine the time-kill kinetics, and the MIC was also calculated as described in a previous publication^18^. From a fresh overnight culture of *E. piscicida*, 1 × 10^6^ colony-forming units (CFU)/mL, 200 µL was transferred to a 96-well plate and treated with different concentrations of LL37, *Ep*EVs, and *Ep*EVs-LL37 (0–40 µg/mL), and the plate was incubated at 30 °C for 24 h. Chloramphenicol (50 µg/mL) was used as the PC. OD_595_ was measured at every 3 h interval using an iMark microplate reader (BIO RAD, Tokyo, Japan) to evaluate bacterial growth. The growth kinetics of *E. piscicida* were depicted in a line graph showing OD_595_ over time. The MIC was identified as the lowest concentration of *Ep*EVs-LL37, which resulted in no change in visual bacterial growth and absorbance compared to the initial OD_595_ value.

To assess the viability of *E. piscicida* following *Ep*EVs-LL37 treatment, an MTT (Sigma-Aldrich, St. Louis, MO, USA) assay was performed based on the method outlined by Jayathilaka et al.^18^. In brief, bacterial cultures (1 × 10^6^ CFU/mL) were exposed to varying concentrations of *Ep*EVs-LL37 and incubated at 30 °C for 24 h. After incubation, the cells were harvested by centrifugation at 1500 × g for 10 min and rinsed with PBS. Subsequently, 20 µL of MTT reagent (5 µg/mL) was added to the cells and incubated for 30 min. Then, 50 µL of dimethyl sulfoxide (DMSO; Sigma Aldrich, Saint Louis, USA) was added to each sample and mixed thoroughly. The OD_595_ was then measured using a microplate reader.

### Morphological changes of *Ep*EVs-LL37-treated *E. piscicida*

To examine the morphological and ultrastructural alterations in *E. piscicida* following *Ep*EVs-LL37 treatment, FE-SEM was carried out with minor adjustments based on a previously described method^18^. *E. piscicida* cultures (1 × 10^6^ CFU/mL) were treated with LL37, *Ep*EVs, and *Ep*EVs-LL37 (40 µg/mL), followed by incubation at 30 °C for 12 h. After incubation, cells were collected by centrifugation at 1500 × g for 10 min, washed with PBS, and pre-fixed with 2.5% glutaraldehyde (Bio-Solution, Gyeonggi-do, Rep. of Korea) for 20 min. The samples were then washed with PBS and dehydrated using a graded ethanol series (30%, 50%, 70%, 80%, 90%, and 100%). Finally, a platinum coating was applied to the bacterial cells using an SP-1ST ion sputter coater (Semian, Daejeon, Rep. of Korea), and the samples were observed using an Ultra High-Resolution versatile FE-SEM SU7000 system (Hitachi High-Tech, Tokyo, Japan).

### Assessment of membrane permeability and oxidative stress in *E. piscicida* upon *Ep*EVs-LL37 treatment

To evaluate changes in membrane permeability and ROS production in *E. piscicida* following *Ep*EVs-LL37 treatment, PI (Sigma-Aldrich, St. Louis, MO, USA) uptake combined with FDA (Sigma-Aldrich, St. Louis, MO, USA) staining, and DCFHDA assays were carried out based on previously established methods^18^. In brief, 2 mL of *E. piscicida* culture (1 × 10^6^ CFU/mL) was treated with LL37, *Ep*EVs, and *Ep*EVs-LL37 (40 µg/mL). PBS served as the NC, while chloramphenicol (50 µg/mL) was used as the PC. The cultures were incubated at 30 °C for 3 h. Cells were then harvested by centrifugation at 1500 × g for 10 min, washed, and resuspended in PBS.

For the PI/FDA uptake assay, the resuspended cells were incubated with PI (50 µg/mL) and FDA (40 µg/mL) at room temperature (26 ± 2 °C) for 30 min in the dark. After staining, excess dyes were removed by washing with PBS, and stained cells were resuspended. From each bacterial cell suspension, 5 µL was placed on glass slides and visualized under an LSM 880 with Airyscan confocal laser scanning microscope (CLSM) system (Carl Zeiss Microscopy, Jena, Germany). Red fluorescence (PI) was detected using excitation and emission wavelengths of 535 and 617 nm, respectively, and green fluorescence (FDA) was measured at 488 and 535 nm, respective excitation and emission wavelengths.

To assess ROS generation, cells were incubated with 50 µg/mL DCFHDA at room temperature (26 ± 2 °C) for 30 min in the dark. After removing excess dye and washing with PBS, green fluorescence, indicative of ROS levels, was visualized under CLSM (after preparing slides as mentioned above) with excitation and emission wavelengths of 488 and 535 nm, respectively.

### Transcriptional regulation of Immunomodulatory genes upon *Ep*EVs-LL37 treatment in vitro and in vivo

 In vitro and in vivo transcriptional regulation of immunomodulatory genes was determined by qRT-PCR as described in our previous study, with minor modifications^16^. Pre-seeded Raw 264.7 cells were treated with LL37, *Ep*EVs, and *Ep*EVs-LL37 (10 µg/mL) and incubated at 37 °C for 24 h. Cells were harvested, and total RNA was isolated using the NucleoSpin RNA Mini Kit (Macherey-Nagel, Duren, Germany), followed by cDNA preparation using the PrimeScript 1st strand cDNA Synthesis Kit (TaKaRa^®^, Shiga, Japan). qRT-PCR was conducted using a Thermal Cycler Dice Real-Time System (TaKaRa^®^, Shiga, Japan) using gene-specific primers (Supplementary Table S1). For in vivo gene expression, 60-hpf zebrafish larvae were treated as mentioned above (20 larvae/replicate; 3 replicates/treatment) for 24 h. Then the larvae were euthanized by a lethal dose of tricaine methanesulfonate (0.2 g/mL) (Sigma-Aldrich, St. Louis, MO, USA) for 2 min. Euthanized larvae were washed with PBS, and excess was removed carefully. Total RNA isolation, cDNA preparation, and qRT-PCR were conducted as mentioned above with zebrafish-specific primers mentioned in Supplementary Table S1. Relative expression fold values were calculated for each gene using the 2^−ΔΔCt^ method^50^. Glyceraldehyde-3-phosphate dehydrogenase (*Gapdh*) and *β actin* were used as housekeeping genes for Raw 264.7 cells and zebrafish larvae, respectively.

### *Ep*EVs-LL37 induced protein expression in vitro and in vivo

Western blotting was performed to determine immune-related protein expression in vitro and in vivo. Raw 264.7 cells and zebrafish larvae (60 hpf) were treated with LL37, *Ep*EVs, and *Ep*EVs-LL37 for 24 h and euthanized as mentioned above. Protein isolation and separation were done after lysing cells and larvae using ProEX™ CeTi Lysis buffer (TransLab, Daejeon, Rep. of Korea). The protein concentration was measured using the Bradford method. Protein (25 µg) from each sample was loaded in 12% sodium dodecyl-sulfate polyacrylamide gel electrophoresis (SDS-PAGE) gels separately (in vitro and in vivo) and electrophorized. Transferred membranes were incubated overnight with specific primary antibodies mentioned in Supplementary Table S2, followed by incubation with secondary antibodies for 1 h. Incubated membranes were visualized using a chemiluminescence imaging system (Fusion Solo S, Vilber, Lourmat, France).

### In vitro cell migration assay

A cell migration assay was carried out using HDF cells (PCS-201-010™, ATCC, Manassas, VA, USA) to assess the wound-healing effects of *Ep*EVs-LL37, based on the method described by Edirisinghe et al.^51^. The cells were cultured in fibroblast basal medium (PCS-201-030™, ATCC, Manassas, VA, USA) supplemented with the Fibroblast Growth Kit–Low Serum (PCS-201-041™, ATCC, Manassas, VA, USA) and maintained at 37 °C, provided with 5% CO_2_ (V/V). HDF cell suspension was seeded at a density of 2 × 10⁵ cells/mL (70 µL per well) into each chamber of a Culture-Insert 2 Well (Ibidi GmbH, Gräfelfing, Germany). Following incubation at 37 °C for 24 h, the inserts were carefully removed to create a 500 μm-wide cell-free gap, mimicking a wound. The culture medium was then replaced with 2 mL of serum-free basal medium (PCS-201-040, ATCC, Manassas, VA, USA) (except for the PC), and LL37, *Ep*EVs, and *Ep*EVs-LL37 were added (5 and 10 µg/mL), respectively, while PBS (100 µL) was added to the NC. Low-serum media (2 mL) was added to the PC. Cell migration into the wound area was observed at 0, 6, 12, 18, and 24 hpt, and images were taken using an inverted microscope (DMi8; Leica, Wetzlar, Germany). The wound area was subsequently quantified using ImageJ software (Ver. 1.8.0, NIH, Bethesda, MD, USA).

### Zebrafish larvae fin regeneration assay

To evaluate the in vivo fin regeneration and wound-healing effects of *Ep*EVs-LL37, a caudal fin regeneration assay was performed. Zebrafish larvae were maintained as described in our previous publication, and those at 60 hpf were selected for caudal fin amputation (*n* = 8 per treatment group)^51^. Anesthesia was induced using 0.05% (w/v) tricaine methanesulfonate (Sigma-Aldrich, St. Louis, MO, USA), and fin amputation was carried out posterior to the notochord using a surgical blade, following a previously published method^51^. After amputation, larvae were transferred to 48-well plates containing embryonic medium. LL37, *Ep*EVs, or *Ep*EVs-LL37 were administered at a concentration of 10 µg/mL, with PBS serving as the NC. Regenerating fins were observed under a light microscope, and images were captured at 0, 2, 4, and 6 dpt. The surface area of regenerating fins was quantified using ImageJ software, and regeneration was assessed by comparing fin areas to those of non-amputated larvae at the corresponding time points.

### Statistical analysis

Statistical analyses for all experiments were conducted using the GraphPad Prism software version 8 (San Diego, CA, USA). A one-way or two-way ANOVA test was used to analyze cell viability, while one-way ANOVA was used for gene expression. In vitro and in vivo wound-healing effect was analyzed using a two-way ANOVA test. Dunnett multiple comparison test was used to compare mean differences, and *p* < 0.05 was considered statistically significant. Triplicate data are expressed as mean ± standard error of the mean (SEM).

## Supplementary Information

Below is the link to the electronic supplementary material.

Supplementary Material 1

## Data Availability

Supporting data used in the study will be available through the corresponding author upon reasonable request.

## Declarations

### Corresponding author

Correspondence to Mahanama De Zoysa.
