# Supplementary material for: Cathelicidin LL37-loaded extracellular vesicles from Edwardsiella piscicida promote antibacterial and wound-healing activity
Source: Sci Rep. 2025 Dec 2;15:45724. doi: 10.1038/s41598-025-28377-9 (PMC12754054; doi:10.1038/s41598-025-28377-9)
Supplement: Supplementary file 1 — Supplementary Material 1 [file 41598_2025_28377_MOESM1_ESM.docx]

Cathelicidin LL37-Loaded Extracellular Vesicles from *Edwardsiella piscicida* Promote Antibacterial and Wound-Healing Activity

Mawalle Kankanamge Hasitha Madhawa Dias, E.H.T. Thulshan Jayathilaka, and Mahanama De Zoysa*

College of Veterinary Medicine and Research Institute of Veterinary Medicine,

Chungnam National University, Yuseong-gu Daejeon 34134, Republic of Korea.

Corresponding author:

*Mahanama De Zoysa (De Zoysa, M)

College of Veterinary Medicine and Research Institute of Veterinary Medicine,

Chungnam National University, Yuseong-gu, Daejeon 34134, Republic of Korea.

E-mail: [mahanama@cnu.ac.kr](mailto:mahanama@cnu.ac.kr)

**Supplementary Table S1**. Details of primers used in the study.

| **Gene name** | **TM (°C)** | **Primer name** | **Primer sequence (5′-3′)** | **Accession no./ Reference** |
| --- | --- | --- | --- | --- |
| **Raw 264.7 cells:** |  |  |  |  |
| Toll-like receptor 2 (*Tlr2*) | 58 | Tlr2-F | CACTATCCGGAGGTTGCATATC | NM_011905 |
|  |  | Tlr2-R | GGAAGACCTTGCTGTTCTCTAC |  |
| Toll-like receptor 4 (*Tlr4*) | 58 | Tlr4-F | GCTTACACCACCTCTCAAACT | NM_021297.3 |
|  |  | Tlr4-R | ACAGCCACCAGATTCTCTAAAC |  |
| Myeloid differentiation primary response factor 88 (*Myd88*) | 58 | Myd88-F | TCGATGCCTTTATCTGCTACTG | NM_010851.3 |
|  |  | Myd88-R | GGTCGGACACACACAACTTA |  |
| Interferon (*Inf*)*α* | 60 | Ifnα-F | CTCTCCTGCCTGAAGGACAGGAAG | NM_010502.2 |
|  |  | Ifnα-R | GGTGGAGGTCATTGCAGAATGAGT |  |
| Interferon (*Inf*)*β* | 60 | Ifnβ-F | TCCAAGAAAGGACGAACATTCG | NM_010510.1 |
|  |  | Ifnβ-R | TGAGGACATCTCCCACGTCAA |  |
| Interleukin 1β (*Il1β*) | 58 | Il1β-F | GGTGTGTGACGTTCCCATTA | NM_008361 |
|  |  | Il1β-R | ATTGAGGTGGAGAGCTTTCAG |  |
| Interleukin 6 (*Il6*) | 58 | Il6-F | CTTCCATCCAGTTGCCTTCT | NM_031168.2 |
|  |  | Il6-R | CTCCGACTTGTGAAGTGGTATAG |  |
| Interleukin 10 (*Il10*) | 58 | Il10-F | TTGAATTCCCTGGGTGAGAAG | NM_010548.2 |
|  |  | Il10-R | TCCACTGCCTTGCTCTTATTT |  |
| Catalase (*Cat*) | 58 | Cat-F | GATGGTAACTGGGATCTTGTGG | NM_009804.2 |
|  |  | Cat-R | GTGGGTTTCTCTTCTGGCTATG |  |
| Superoxide dismutase 1 (*Sod1*) | 58 | Sod1-F | GGTTCCACGTCCATCAGTATG | NM_011434.2 |
|  |  | Sod1-R | GTCTCCAACATGCCTCTCTTC |  |
| Glyceraldehyde-3-phosphate  dehydrogenase (*Gapdh*) | 56 | Gapdh-F | AGGTCATCCCAGAGCTGAACG | NM_001289726.1 |
|  |  | Gapdh-R | CACCCTGTTGCTGTAGCCGTAT |  |
| **Zebrafish:** |  |  |  |  |
| Toll-like receptor 2 (*tlr2*) | 58 | tlr2-F | TCTCCGTCTTGGTTTCAC | NM_212812.1 |
|  |  | tlr2-R | GGTCCCACAGTTGAGTATG |  |
| Toll-like receptor 4 (*tlr4*) | 58 | tlr4b-F | GGAATAATGGGCAGCCGTAAG | AY388400.1 |
|  |  | tlr4b-R | AGCGACACCAGGAACTATCAATG |  |
| Toll-like receptor 5b (*tlr5b*) | 56 | tlr5b-F | GAAACATTCACCCTGGCACA | BC163185.1 |
|  |  | tlr5b-R | CTACAACCAGCACCACCAGAATG |  |
| Interleukin-1β (*il1β*) | 58 | il1β-F | TCAAACCCCAATCCACAGAG | AY340959.1 |
|  |  | il1β-R | TCACTTCACGCTCTTGGATG |  |
| Interleukin (*Il8*) | 57 | il8-F | CTTCCCTCCAAGCCCACAC | XM_009306855.2 |
|  |  | il8-R | GATCCGGGCATTCATGG |  |
| Interleukin-10 (*il10*) | 58 | il10-F | CCCTATGGATGTCACGTCATG | AY887900.1 |
|  |  | il10-R | CATATCCCGCTTGAGTTCCTG |  |
| Tumor necrosis factor- α (*tnfα*) | 58 | tnfα-F  tnfα-R | AGAAGGAGAGTTGCCTTTACCGCT | AY427649 |
|  |  |  | AACACCCTCCATACACCCGACTTT |  |
| Myeloid differentiation primary response factor 88 (*myd88*) | 56 | myd88-F | AACAACTTCGCTGGATAA | DQ100359.1 |
|  |  | myd88-R | GTTACTGGAATCGCCTCA |  |
| Superoxide dismutase 1 (*sod1*) | 57 | sod1-F | AGGTGACTGGTGAAATTACTGG | NM_131294.1 |
|  |  | sod1-R | GTCTCACACTATCGGTTGGC |  |
| Beta actin (*β actin*) | 58 | β actin- F | AATCTTGCGGTATCCACGAGACCA | AF025305 |
|  |  | β actin- R | TCTCCTTCTGCATCCTGTCAGCAA |  |

**Supplementary Table S2**. Details of antibodies used in the study.

| **Primary antibody** | **Manufacturer/Reference No.** | **Dilution** | **Secondary antibody** | **Manufacturer/Reference No.** | **Dilution** |
| --- | --- | --- | --- | --- | --- |
| Heat shock protein 90 (Hsp90) | Cell Signaling Technology, MA, USA (4874) | 1:1000 | Anti-rabbit IgG-HRP | Cell Signaling Technology, MA, USA (7074) | 1:3000 |
| Heat shock protein 70 (Hsp70) | Cell Signaling Technology, MA, USA (4872) | 1:1000 | Anti-rabbit IgG-HRP | Cell Signaling Technology, MA, USA (7074) | 1:3000 |
| Nuclear factor κB p65 subunit (p65 NF-κB) | Cell Signaling Technology, MA, USA (4764) | 1:1000 | Anti-rabbit IgG-HRP | Cell Signaling Technology, MA, USA (7074) | 1:3000 |
| Transforming growth factor-beta 1 (Tgfβ1) | Antibodies-online GmbH, Aachen, Germany (ABIN6711930) | 1:1000 | Anti-rabbit IgG-HRP | Cell Signaling Technology, MA, USA (7074) | 1:3000 |
| Interleukin 10 (Il10) | Kingfisher Biotech, Inc., MI, USA (KP1267Z-100) | 1:1000 | Anti-rabbit IgG-HRP | Cell Signaling Technology, MA, USA (7074) | 1:3000 |
| Interferon gamma (Ifnγ) | Kingfisher Biotech, Inc., MI, USA (KP1233Z-100) | 1:1000 | Anti-rabbit IgG-HRP | Cell Signaling Technology, MA, USA (7074) | 1:3000 |
| Tumor necrosis factor α (Tnfα) | Kingfisher Biotech, Inc., MI, USA (KP1540Z-100) | 1:1000 | Anti-rabbit IgG-HRP | Cell Signaling Technology, MA, USA (7074) | 1:3000 |
| Βeta actin (β actin) | Santa Cruz Biology Inc., OR, USA (sc-4778) | 1:1000 | Anti-mouse IgG-HRP | GeneTex, Quebec, Canada  (GTX213111-01) | 1:3000 |


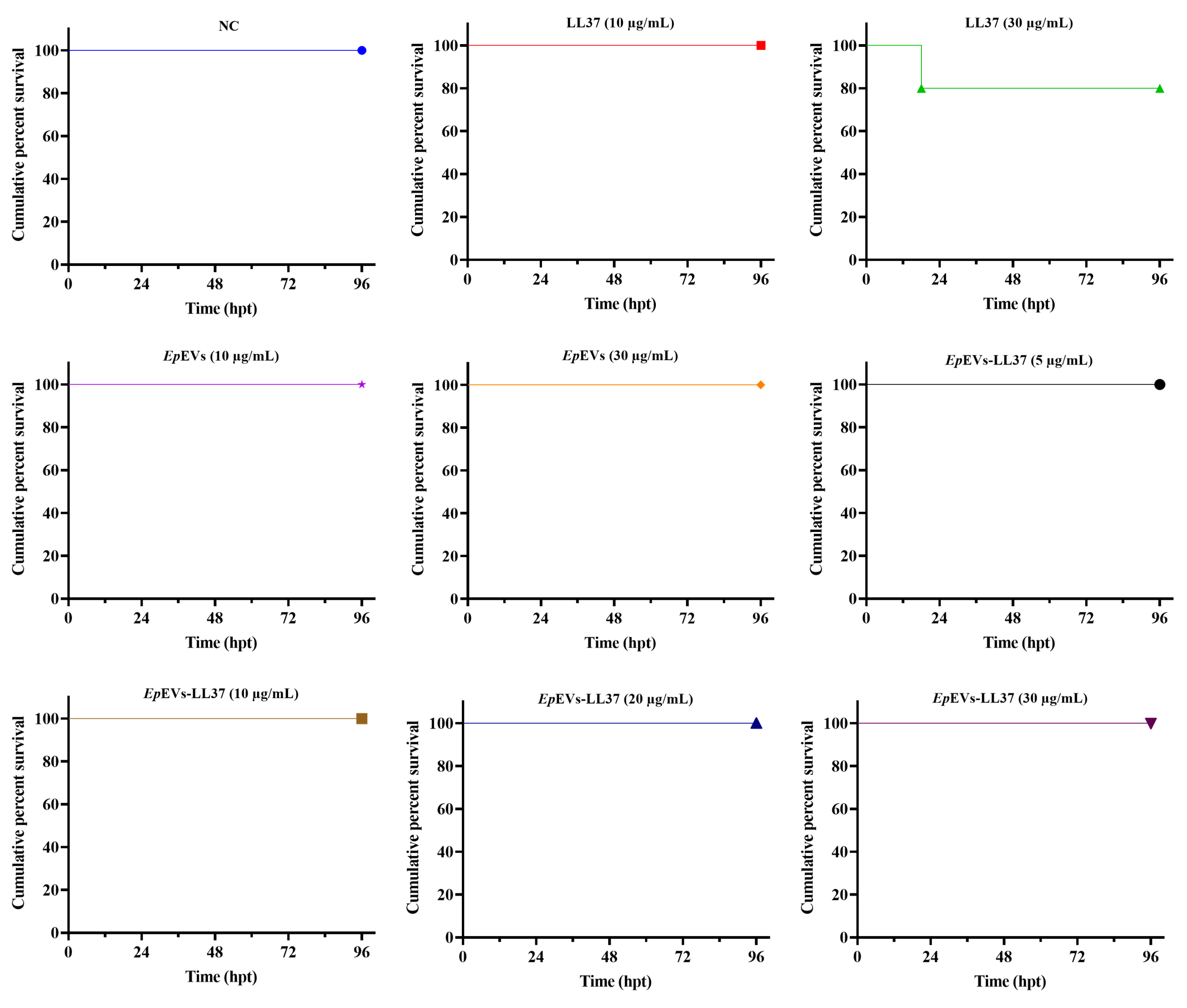


**Supplementary Fig. S1.** *In vivo* toxicity determination for each treatment using zebrafish larvae. Mortality of *Ep*EVs-LL37 (0-30 µg/mL) pre-treated zebrafish larvae was observed until 96 h post-treatment (hpt), and the cumulative percent survival was individually graphed with time.

**
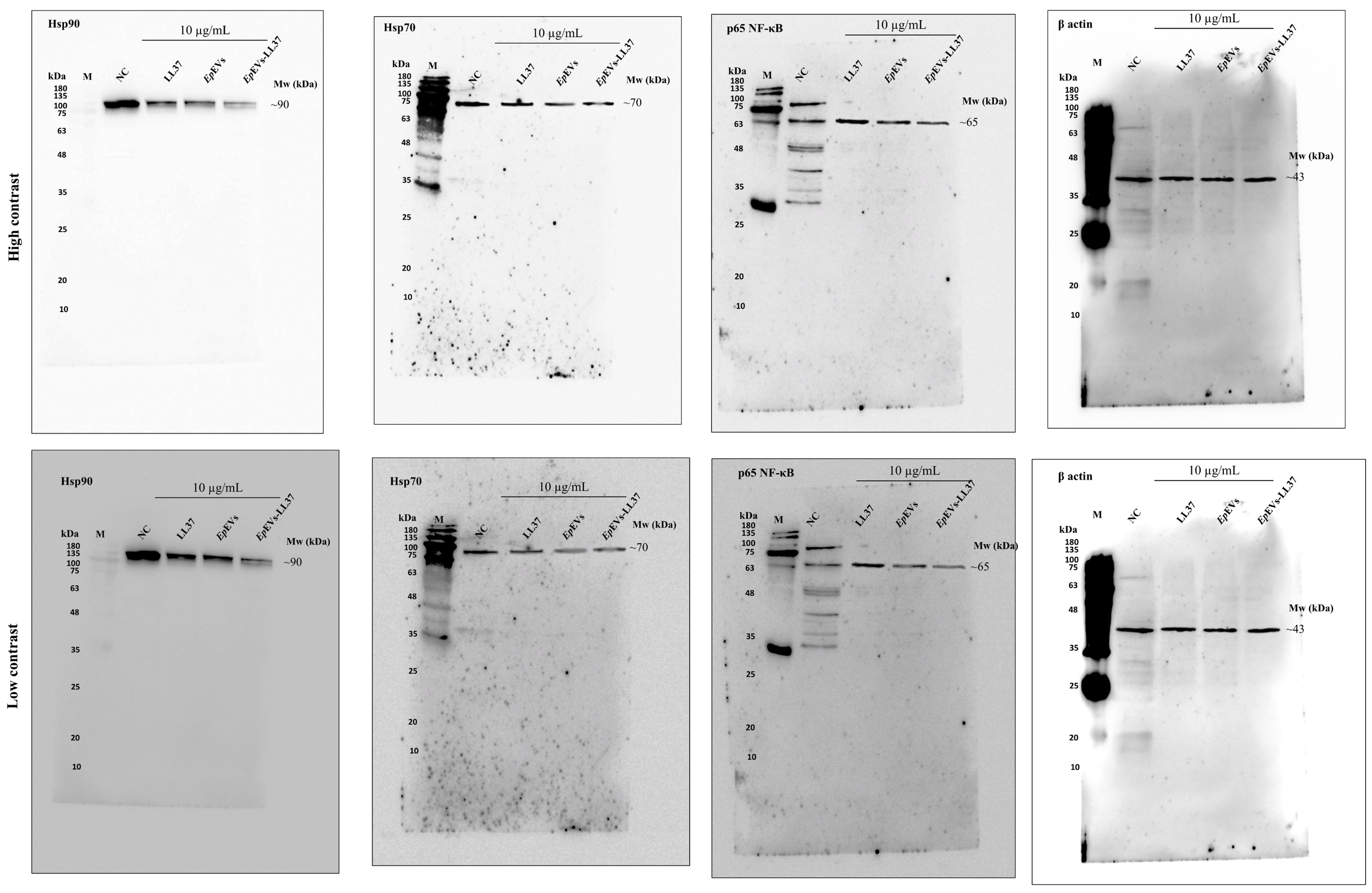
**

**Supplementary Fig. S2**. Uncropped full blots of the i*n vitro* immunomodulatory protein expression of *Ep*EVs-LL37 in Raw 264.7 cells at different contrast levels. The protein marker molecular weight is also provided on the left. (M = marker)


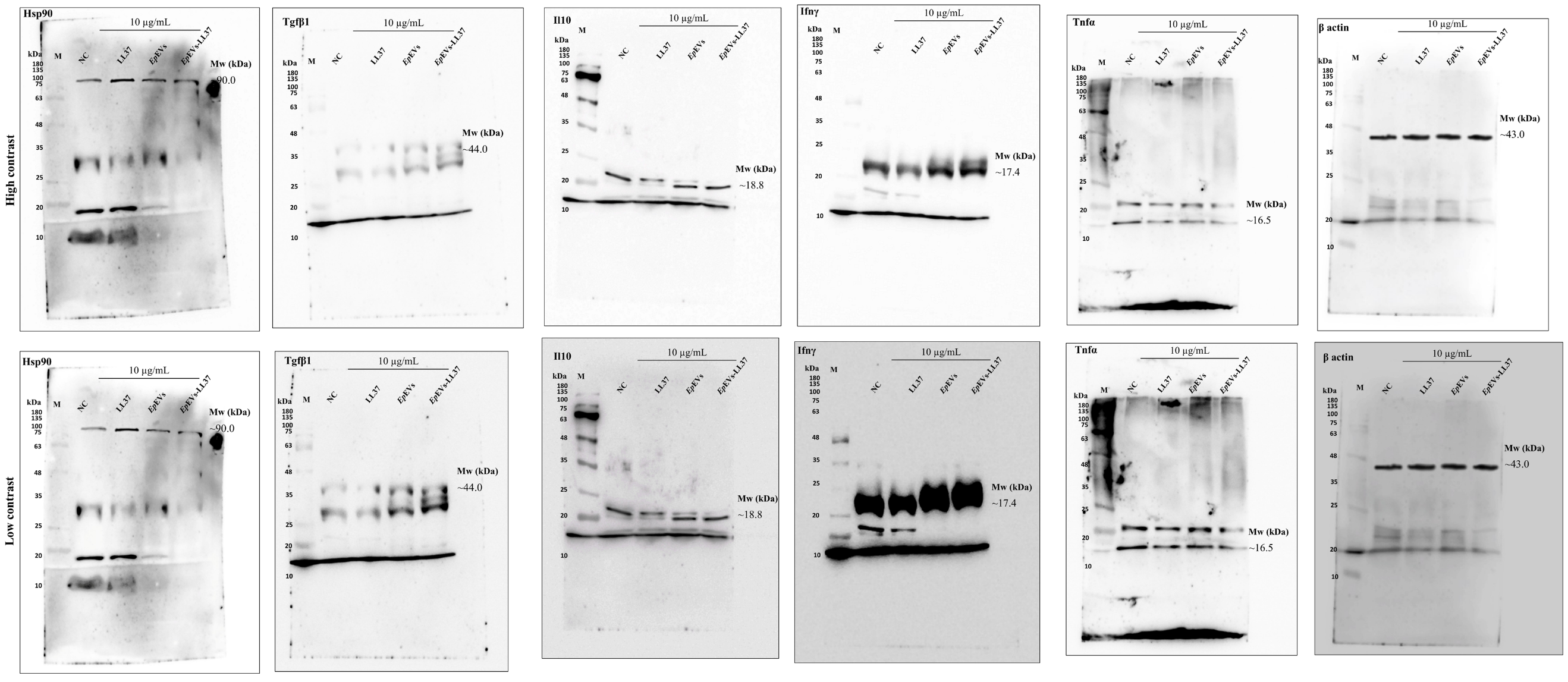


**Supplementary Fig. S3**. Uncropped full blots of the i*n vitro* immunomodulatory protein expression of *Ep*EVs-LL37 in zebrafish larvae (60 hpf) at different contrast levels. The protein marker molecular weight is also provided on the left. (M = marker)
